# Quantitative Insights into the Contribution of Nematocysts to the Adaptive Success of Cnidarians Based on Proteomic Analysis

**DOI:** 10.3390/biology11010091

**Published:** 2022-01-07

**Authors:** Qingxiang Guo, Christopher M. Whipps, Yanhua Zhai, Dan Li, Zemao Gu

**Affiliations:** 1 Department of Aquatic Animal Medicine, College of Fisheries, Huazhong Agricultural University, Wuhan 430070, China; qingxiang.guo@outlook.com (Q.G.); zhaiyh@mail.hzau.edu.cn (Y.Z.); liidan@webmail.hzau.edu.cn (D.L.); 2 Hubei Engineering Technology Research Center for Aquatic Animal Diseases Control and Prevention, Wuhan 430070, China; 3 Engineering Research Center of Green Development for Conventional Aquatic Biological Industry in the Yangtze River Economic Belt, Ministry of Education, Wuhan 430070, China; 4 SUNY-ESF, College of Environmental Science and Forestry, State University of New York, 246 Illick Hall, 1 Forestry Drive, Syracuse, NY 13210, USA; cwhipps@esf.edu

**Keywords:** adaptive evolution, phenotypic novelty, cnidarians, myxozoans, nematocysts, toxin

## Abstract

**Simple Summary:**

Cnidarians (such as corals, anemones, jellyfish) are ancient and successful animals. Previous studies have offered a variety of explanations for the adaptive success of certain cnidarian taxa. However, common strategies for the long-term persistence of cnidarians have not been identified. One factor that may contribute to their evolutionary success of the lineage is the nematocyst, a sting organelle able to deliver venom into prey or enemies. Using bioinformatics analyses, we aimed to quantitatively investigate the role of nematocysts in cnidarian adaptation. We identified the extensive species-specific adaptation in nematocyst proteins (NEMs) and demonstrate that both a unique evolutionary pattern of NEMs and the long evolutionary lag between nematocysts and cnidarians support their key adaptive role. Further, we find NEMs experience approximately 50% more adaptive changes on average compared to non-NEMs, and positively selected cnidarian-conserved proteins are enriched in NEMs. These results support a key role of nematocysts in successful cnidarian adaptation and provide a general quantitative framework for assessing the role of a phenotypic novelty in adaptation. Moreover, the findings will be critical for reassessing the evolutionary history of many established models, enhancing our understanding of both the mechanisms and evolutionary preference of adaptive evolution.

**Abstract:**

Nematocysts are secretory organelles in cnidarians that play important roles in predation, defense, locomotion, and host invasion. However, the extent to which nematocysts contribute to adaptation and the mechanisms underlying nematocyst evolution are unclear. Here, we investigated the role of the nematocyst in cnidarian evolution based on eight nematocyst proteomes and 110 cnidarian transcriptomes/genomes. We detected extensive species-specific adaptive mutations in nematocyst proteins (NEMs) and evidence for decentralized evolution, in which most evolutionary events involved non-core NEMs, reflecting the rapid diversification of NEMs in cnidarians. Moreover, there was a 33–55 million year macroevolutionary lag between nematocyst evolution and the main phases of cnidarian diversification, suggesting that the nematocyst can act as a driving force in evolution. Quantitative analysis revealed an excess of adaptive changes in NEMs and enrichment for positively selected conserved NEMs. Together, these findings suggest that nematocysts may be key to the adaptive success of cnidarians and provide a reference for quantitative analyses of the roles of phenotypic novelties in adaptation.

## 1. Introduction

Cnidarians, a group of invertebrates, have survived five mass extinctions and have persisted for more than 700 million years [1,2]. Cnidarians have a simple body plan but diverse morphologies and complex life cycles [3]. They include more than 12,400 species, both free-living and parasitic, and are distributed in nearly all aquatic [3] and even terrestrial habitats [4]. Previous studies have proposed gene- or environment-based mechanisms to explain the adaptive success of certain cnidarian taxa [2,5]. However, common strategies for the long-term persistence of cnidarians have not been identified. One factor that may contribute to the evolutionary success of the lineage is the nematocyst, a secretory organelle able to deliver chemical arsenals into prey or predators [6,7].

The nematocyst is a phenotypic novelty in cnidarians, observed in fossils from the Middle Cambrian period [1,8]. Each nematocyst consists of a capsule and a coiled tubule through which venom is injected with an ultrafast acceleration of 5 million g [9,10]. The venom induces paralysis for prey capture and may cause pain, possibly for predator deterrence [11]. Nematocysts are also used for locomotion in *Hydra* and host attachment in parasitic myxozoans [7,12]. Strong selective pressures are expected to result in an arms race. However, it is difficult to quantitatively evaluate the extent to which the nematocyst contributes to adaptation and to explore the molecular mechanisms underlying this trait, in part because the trait is polygenic, making it challenging to quantitatively detect selection acting on multiple genes simultaneously [13]. Previous studies of novelties have focused on a few genes [14] or qualitatively identified a trait as a key innovation by linking it to increased diversification rates [15,16]. This approach may ignore environmental factors and may result in the identification of a complex evolutionary process as a single event [17]. Until now, the role of the nematocyst in cnidarian adaptive evolution and associated evolutionary processes are unclear. Resolving these issues requires knowledge of the specific set of genes that encode the nematocyst proteome. These genes might reveal a unique signature associated with nematocyst evolution and provide a basis for identifying candidate components proximal to nematocyst composition and function [18].

The nematocyst proteomes of several cnidarians have recently been published, including proteomes of some anthozoans [19,20,21,22], hydrozoans [18,21,22,23] cubozoans [24,25], scyphozoans [21,25,26,27,28,29,30,31], and a myxozoan [32]. These pioneering studies have provided important insights into the evolutionary history of the nematocyst (for example, [9,21,33]). Nonetheless, we still lack a clear perspective on the role of this organelle in the adaptive success of cnidarians over the past 700 million years.

Myxozoa are microscopic, oligocellular endoparasites with simple body organization, but multiple morphologies in their complex life cycles [34]. They have only recently been classified as cnidarians and, together with *Polypodium hydriforme*, form a sister clade to Medusozoa [3,35]. Myxozoans are the only parasitic group that entailed substantial radiation in cnidarians and represent about 20% of cnidarian species diversity [36]. Myxospores consist of 1–13 polar capsules (referred to as nematocysts below) that have a functionality different from that of free-living species–attaching the host [37,38]. Despite lacking barbs or spines, the discharged tubules of some myxozoans have retained the ability to inject chemicals and other structural elasticity not found in tubules of free-living cnidarians [12,39,40,41]. Thus, proteomic data of myxozoans are an important addition to understanding the adaptive evolution of cnidarian nematocysts. However, there is only one study to our knowledge on the myxozoan nematocyst proteome, which performed a comprehensive functional and proteomic analysis of *Ceratonova shasta* [32].

Here, we used three newly generated proteomes for myxozoan nematocysts in conjunction with five previously published proteomes and 110 cnidarian transcriptomes/genomes to determine the extent of positive selection in nematocyst proteins (NEMs) and to characterize their evolution in detail. We detected extensive species-specific adaptations in NEMs as well as evidence for decentralized evolution, in which most evolutionary events involved a non-core NEM set, possibly due to the rapid diversification of NEMs in cnidarians. Additionally, we detected a macroevolutionary lag between nematocyst evolution and the main phases of cnidarian diversification, suggesting that the nematocyst was an evolutionary driving force. This was supported by quantitative analyses of relative adaptation and enrichment of NEMs against reference proteins. We conclude that the nematocyst may be a key determinant of the adaptive success of cnidarians by conferring flexibility (evolvability) and therefore by promoting adaptation to changing selection regimes. Our results provide new insights into the success of cnidarians and build a foundation for quantifying the contribution of a phenotypic novelty to evolutionary success.

## 2. Materials and Methods

### 2.1. Collecting Details

*Myxobolus honghuensis* was collected from infected allogynogenetic gibel carp *Carassius auratus gibelio* in Zoumaling Farm, Hubei Province, China on 29 July 2015. *Myxobolus wulii* was sampled from infected allogynogenetic gibel carp in Yellow Sand Port, Jiangsu Province, China on 8 August 2016. *Thelohanellus kitauei* was collected from infected common carp *Cyprinus carpio* in Liuerbao Town, Shenyang Province, China on 11 August 2015. Fish were held on ice before being killed with an overdose of MS-222 (Sigma–Aldrich, Co., Ltd., St. Louis, MO, USA). From each fish, tissue containing one large cyst was homogenized by a manual glass tissue grinder and suspended in 0.1 M phosphate-buffered saline (PBS), pH 7.2, and then filtered through cotton gauze. Myxospores were separated from the filtrate by sucrose gradient centrifugation and Percoll gradient centrifugation in turn, washed several times with distilled water, and then examined microscopically to verify purity and identify. Purified myxospores were either placed into RNAlater (Sigma), frozen in liquid nitrogen and finally stored at −80 °C, or immediately sent to nematocyst isolation as described below. Myxozoan identification was performed based on morphology and 18S sequencing [42,43]. The maintenance and care of experimental animals complied with the National Institutes of Health Guide for the care and use of laboratory animals [44], approved by the animal care and use committee of Huazhong Agricultural University, China (HZAUFI-2015-011).

### 2.2. Nematocyst Isolation and Purity/Integrity Verification

Myxozoan nematocysts were isolated using our previously described methods [45]. Purified myxospores were disrupted by sonication for 3 min (on for 3 s and rest for 1 s) at 85 W electric power. Thereafter, by successively using 50%, 70%, 90% Percoll gradient centrifugation, and 100% Percoll centrifugation, pure nematocysts (50%/70% interface and the middle of the 70% layer) were separated from the sonicated suspension. The nematocysts were intact and pure from contaminating cellular debris based on light microscopy. To provide further evidence of the purity of the nematocysts, we fixed the *T. kitauei* nematocysts in 3% glutaraldehyde at 4 °C, dehydrated with CO_2_ using the critical point method and sputter-coated with gold. The microstructure was characterized with a Japanese SU8010 field emission scanning electron microscope (FESEM) at 3 kV.

### 2.3. Next-Generation Sequencing and Assembly

We generated novel RNA-seq data for seven myxozoans and genomic data for two myxozoans (Table S1). Total RNA was extracted from frozen preserved specimens using TRIzol 550 (Invitrogen). The purity and integrity of RNA were assessed using a NanoPhotometer^®^ spectrophotometer (IMPLEN, Westlake Village, CA, USA) and RNA Nano 6000 Assay Kit from the Agilent Bioanalyzer 2100 system (Agilent Technologies, Santa Clara, CA, USA), respectively. Libraries were prepared from the purified mRNA using a NEBNext^®^ Ultra RNA kit (New England Biolab (NEB), Ipswich, MA, USA) following manufacturer’s recommendations and sequenced as 2 × 125 paired-end (PE) runs with Illumina HiSeq 2500 for *M. honghuensis*, *T. kitauei*; as 2 × 150 paired-end runs with Illumina HiSeq 4000 for *Henneguya yanchengensis*, *Myxobolus ampullicapsulatus*, *M. honghuensis*, *Myxobolus turpisrotundus*, *M. wulii*, *Myxobolus xiantaoensis*, and *T. kitauei*. Raw sequence data were cleaned and trimmed by removing adaptor and low-quality reads using Trimmomatic v0.33 [46]. Filtered reads were de novo assembled through Trinity v2.6.6 [47] and clustered using CD-HIT v4.6.8 [48] and TGICL v2.1 [49].

For genome sequencing, DNA was extracted from the frozen spores of *M. honghuensis* and *M. wulii* using a TIANamp Genomic DNA kit (TIANGEN Inc., Beijing, China). Libraries were built using the TruSeq DNA library kit (Illumina). The ~500 bp (*M. honghuensis*) and ~450 bp (*M. wulii*) insert libraries were sequenced on an Illumina HiSeq 2500 (2 × 125 PE) and Illumina HiSeq 4000 (2 × 150 PE), respectively. After filtering with Trimmomatic v0.33, 19.6 Gb and 18.5 Gb clean data were generated for *M. honghuensis* and *M. wulii* and were assembled with SOAPdenovo v2.04 using default parameters [50]. As the genomic and transcriptomic data were to be used specifically for subsequent MS/MS and phylogenomic analyses, we did not perform further systemic post-assembly analyses in this study.

### 2.4. Data Decontamination and Construction Databases for MS/MS Analysis

We used our recently developed method to construct a clean, efficient, and comprehensive protein reference that we denote as the customized comprehensive proteomic reference database (CCPRD) (for details, see [51]). Briefly, nonredundant host databases containing proteins or nucleotide sequences from the host, as well as nonredundant closely related databases containing proteins or nucleotide sequences from species closely related to myxozoans, were constructed. These four databases were then blasted against the transcriptome assemblies using TBLASTN or TBLASTX in BLAST + v2.4.0 [52] (−*e* value 1 × 10^−5^). Only transcriptome sequences that exclusively matched the host databases were removed. The retained transcriptome sequences were further blasted against a bacterial database using BLASTX (−*e* value 1 × 10^−10^). The positive hits were blasted to the NCBI nonredundant protein database (NR) using BLASTX (−*e* value 1 × 10^−5^). Only sequences that were annotated as “bacteria” were removed. For assessment and visualization of contamination in genomes, taxonomic assignment of each contig was carried out using Blobtools v1.0 [53]. Sequences that strongly matched Chordata and Proteobacteria were excluded. The decontamination process was operated conservatively to prevent possible over-decontamination that could result in the loss of a large portion of actually expressed genes and proteins.

The CCPRDs of *M. honghuensis*, *M. wulii*, and *T. kitauei* were built by integrating information from the genomic and RNA-seq data. For genomes, we used GeneMark-ET [54], Augustus [55], and Semi-HMM-based Nucleic Acid Parser (SNAP) [56] for ab initio prediction, Genewise [57] for homology-based prediction, and PASA [58] for transcriptome-assisted prediction. For RNA-seq data, coding sequences were first predicted by TransDecoder [59] and GeneMarkS-T [60]. Next, transcriptome homology-based predictions were performed by a customized Perl script Hercules (https://github.com/qingxiangguo/hercules) (accessed date: 29 March 2017). Furthermore, those transcripts that were translated neither by de novo nor homology-based method were translated into amino acid sequences using the Transeq script from the European Molecular Biology Open Software Suite (EMBOSS) [61]. Finally, all proteins (>30 amino acids) resulting from genomic and RNA-seq data were processed by CD-HIT with a threshold of 100% to collapse the group into a nonredundant data set, leading to the final CCPRD.

### 2.5. Tandem Mass Spectrometry and Proteomic Analysis

Proteins were extracted from the cleaned nematocysts by dissolving them in lysis buffer (4% SDS, 100 mM Tris-HCl pH 7.6, 1 mM DTT). Protein samples (20 μg) were separated on 10% SDS-polyacrylamide gel electrophoresis and visualized with Coomassie Blue R-250 staining (Figure S1). A 60 μL protein solution was collected, and DTT was added to a 10 mM final concentration, heated at 60 °C for 30 min, and then cooled to room temperature. Then, 200 μL UA buffer (8 M urea, 150 mM Tris-HCl, pH 8.0) was added and centrifuged at 14,000× *g* for 15 min two times; 100 μL IAA buffer (100 mM iodoacetamide in UA) was added and vortexed at 600 rpm for 1 min. The samples were incubated for 30 min in darkness and were centrifuged at 14,000× *g* for 15 min. Then, 100 μL UA buffer was added and centrifuged at 14,000× *g* for 15 min two times, after which 100 μL NH_4_HCO_3_ buffer (25 mM) was added and centrifuged at 14,000× *g* for 15 min two times. Finally, the protein suspensions were digested with 40 μL Trypsin buffer (2 μg Trypsin in 40 μL 100 mM NH_4_HCO_3_) and vortexed (600 rpm, 1 min) before incubation at 37 °C overnight, and the resulting peptides were collected as a filtrate. The filtrated peptides of each sample were desalted on C18 cartridges (Sigma, St. Louis, MO, USA) and were concentrated by vacuum centrifugation and reconstituted in 40 µL of 0.1% (*v*/*v*) formic acid. The peptides were eluted with a linear 110 min gradient of 8–45% and 10 min to 95% acetonitrile with 0.1% formic acid in water at a flow rate of 0.25 μL/min. The samples were analyzed using an Easy-nLC nanoflow HPLC system connected to a Q-Exactive mass spectrometer (Thermo Fisher Scientific, San Jose, CA, USA). MS/MS scans were acquired using Q-Exactive with a mass resolution of 70,000 and 17,500, respectively. The MS scan range was 300–1800 *m*/*z* with automatic gain control (AGC) target 3.0 × 10^−6^, and the maximum injection time was 50 ms. The AGC target and the maximum injection time for MS/MS were 3.0 × 10^−6^ and 60 ms, respectively. The higher-energy collisional dissociation (HCD) mode was used as the fragmentation mode with 27 eV collision energy. The precursor isolation windows were set to 2 *m*/*z*. The raw files were searched against the CCPRD using MaxQuant v1.5.3.8 [62]. The search was set at a precursor mass window of 6 ppm and a fragment tolerance of 20 ppm. The enzyme was set to trypsin. Missed cleavage sites were set to 2. For all searches, carbamidomethylated cysteine was set as a fixed modification and oxidation of methionine and N-terminal protein acetylation as variable modifications. The false-discovery rate for peptides and proteins was set to 0.01. To estimate protein abundance, we used the iBAQ (intensity-based absolute quantification) [63] option of MaxQuant.

As a single peptide can be linked to multiple proteins (e.g., in the case of razor proteins), multiple protein IDs can be assigned to the same protein groups. To prevent the interference of redundant proteins in downstream analysis while still retaining maximum information, a leading protein-based protein identification strategy was introduced here. Briefly, an initial table containing the protein group information from three replicates was constructed. After excluding the proteins marked with “only identified by site”, “reverse”, and “contaminant” from the initial table, leading proteins and majority proteins from all three replicates were merged. The proteins (leading and majority) were annotated with the NR database, and leading protein sequences that needed correction were detected and marked with red color (Dataset S1). Comprehensive contaminants were manually determined with a holistic consideration of blast results, iBAQ, and unique peptide counts. Gene Ontology (GO) analysis and domain predictions were performed for the leading proteins with the BLAST2GO tool v4.19 [64] and InterPro [65], respectively. The final table was improved by correcting the leading protein sequences, removing the comprehensive contaminants and recalculating the sequence length and iBAQ%. To evaluate the repeatability within replicates and the consistency between leading and majority proteins, polar capsule proteins identified in different selection criteria in three replicates were compared. The Venn diagram in Figure S2 indicated good repeatability within replicates and that downstream analysis based on leading proteins had good representativeness for that of majority proteins. The identified proteins (accession numbers, sequences, Gene Ontology (GO) annotations, and InterPro results) are summarized in Dataset S1.

### 2.6. Comparative Proteomic and Venomic Analyses

We identified orthologs using OrthoMCL v2.0.9, which applies the Markov clustering algorithm [66]. Default options were used in all steps for nematocyst proteomes from eight species, namely, myxozoans *M. honghuensis*, *M. wulii*, *T. kitauei*, and *C. shasta* [32], and the free-living cnidarians *Hydra vulgaris*, *Anemonia viridis*, *Aurelia aurita* [21], and *C. fleckeri* [24]. To compare functionalities, BLAST2GO v4.19 [64] was used to obtain the InterPro domains found in the various NEMs, with comparisons performed using Microsoft Excel. For both orthologs and domains, the intra-group comparisons of the myxozoans were visualized in R package VennDiagram [67], while the results from the eight nematocyst proteomes were visualized in UpSetR [68]. A comparison between the myxozoan nematocyst proteomes and the four free-living cnidarian nematocyst proteomes was carried out using BLASTp (−*e* value 1 × 10^−20^). Circoletto [69] and Circos [70] were used to represent the BLASTp results.

To identify the putative toxins, a group of known cnidarian toxin sequences (e.g., metalloprotease, ShK, MAC-PF domains) was used as seed for reciprocal best BLAST hit (RBBH) analysis with nematocyst proteomes (−*e* value 1 × 10^−10^). Then, a machine learning-based classifier ToxClassifier [71] was used to exclude the proteins with non-toxic physiological functions. All candidate toxin proteins identified by RBBH or ToxClassifier were validated by blasting against Tox-Prot UniProtKB/Swiss-Prot [72], and only proteins with the best match to toxins were retained (−*e* value 1 × 10^−5^). To generate an overview of the distribution pattern of putative toxin protein families across cnidarians, a matrix of the presence and absence of toxins in the present study and 23 well-annotated nematocyst proteomes [18,19,20,21,22,23,24,25,26,27,28,29,30,31,32] was constructed (Dataset S2) and visualized. To avoid dramatically increasing the number of potential toxins that could be explored, proteomes from whole tissues (e.g., tenacles, mucus) were not included. The toxin information was extracted directly from those original studies.

### 2.7. Ortholog Assessment, Gene Selection, and Data Filtering

For the cnidarian phylogeny dataset, a new supermatrix was assembled from 110 cnidarian transcriptomes and/or genomes, including our newly generated myxozoan data (Dataset S3). HaMStR v13.2.3 [73] was used to determine orthologous genes following the protocol in Reference [74]. Translated genes for all taxa were searched against the basal model organism core ortholog set of HaMStR using the strict option and *Nematostella vectensis* as the reference taxon. The orthologous groups were then aligned using MAFFT v7.205, and ambiguously aligned regions were trimmed with trimAL v1.4.3 [75]. Alignments that covered fewer than 50 taxa were deleted. Filtering of paralogs was performed in PhyloTreePruner v1.0 [76] based on trees generated with FastTree v2.1.11 [77] using default settings. The protein alignments were concatenated into an initial cnidarian matrix of 51,598 amino acid positions across 146 genes with 103 taxa. TreSpEx v1.1 [78] was used to detect possible effects of sequence biases (LBA and saturation) in our phylogenomic data. LB scores and pairwise distances [78] were calculated for each taxon and OG. Following Struck [78], these values were plotted in R, and outliers were identified as taxa or genes that could cause LBA artifacts or saturation. In addition, taxon-specific LB scores for each gene were calculated, and a heatmap of these scores was generated to identify LBA in a specific taxon (LB score higher than 200). To identify exogenous contamination missed by our initial orthology inference approach, we blasted the alignments against a database that included a set of representative cnidarian sequences and a set of representative bilaterian sequences. Then, we removed all sequences that had better BLAST hits to bilaterians than to cnidarians. Finally, a series of pruning experiments were conducted by removing those taxa and genes identified to be LBA artifacts, saturated, and contaminations. The outline for phylogenomic analysis is summarized in Figure S3.

For the nematocyst phylogeny, nematocyst core proteins were identified by OrthoMCL v2.0.9 from eight nematocyst proteomes and were used as queries to search annotated proteins from cnidarian transcriptomes or genomes with BLASTp. Reciprocal best hits recovered at a cutoff of 1 × 10^−5^ among the cnidarian proteins and the matched sequences were considered to be orthologs. As a secondary check, HMMER v3.0 [79] was also used to find potential homologs. Retrieved amino acid sequences were aligned with MAFFT v7.205 [80] by the L-INS-i method. Multiple alignments were trimmed using Gblocks v0.91b [81]. In cases where there were multiple copies of homologs of a particular gene, single-gene trees were built using IQ-TREE v1.6.4 [82] and were then manually inspected to remove suspicious sequences and paralogs using FigTree v1.4.3 (http://tree.bio.ed.ac.uk/software/figtree/) (accessed date: 5 November 2017). Single-gene alignments were concatenated using SCaFoS v1.2.5 [83].

### 2.8. Phylogenetic Analysis

The phylogenomic and nematocyst datasets were analyzed with both ML and Bayesian inference (BI) methods. The ML analyses were conducted using RAxML v.8.2.12 [84] and IQ-TREE v1.6.4 [82]. Nonparametric ML bootstrap support was obtained from 100 ML replicates using the PROTGAMMAAUTO model implemented in RAxML. The IQ-TREE-inferred ML phylogeny used the LG + C20 + F + Γ4 model plus 10,000 ultrafast (UF) bootstrap approximations. Bayesian Inference was performed with PhyloBayes MPI v1.4 [85] using the CAT + GTR + Γ4 model. We ran four Markov chain Monte Carlo (MCMC chains for 20,000 generations and saved every second tree. We tested for convergence (maxdiff < 0.1) using bpcomp, implemented in PhyloBayes, with a burn-in of 20%.

Approximately unbiased [86] tests of various competing phylogenetic hypotheses were conducted using loose constraints with the hypothetical groupings and optimizing these constraints under LG +Γ4 + F + C20 + PSMF in IQ-TREE using the 146-gene (51,598 amino acid positions) dataset. The optimized trees were compared using the approximately unbiased test with 10,000 RELL bootstrap replicates. Maximum log-likelihoods of each constraint and their differences from the optimal ML tree were calculated and compared. The hypotheses within the 95% confidence interval that could not be rejected were where *p* > = 0.05.

To assess if fast-evolving genes were biasing inferences [87], we estimated the rates of evolution per site with Dist_Est [88] under the LG + Γ4 model and removed the fastest evolving sites in a stepwise fashion (3500 sites per step). Each step was analyzed using 10,000 MLBS pseudoreplicates in IQ-TREE under LG + Γ4 + F or LG +Γ4 + F + C20 + PSMF.

### 2.9. Divergence Time Estimation

The divergence times of nematocyst core proteins and the major cnidarian lineages were calculated across the best ML tree, which was generated by RAxML, using Penalized likelihood (PL) implemented in r8s v1.7.1 [89]. Cross-validation was tested to determine the best smoothing value, and the smoothing parameters (cvstart = −8; cvinc = 1; cvnum = 15) were set to 1 × 10^−6^. Four calibration points were used: the node of crown cnidarians was constrained with a maximum age of 741 Mya (million years ago) [90], the minimum age of Medusozoa was set to 570 Mya [91], the minimum age of Hexacorallia was set to 540 Mya [90], and the rise of Hydrozoa was fixed at 500 Mya [8].

### 2.10. Selection Analyses and Adaptation Quantification

Selection analysis was performed using Hyphy v2.3.14 [92]. For individual toxins (astacin, C-type lectin, disintegrin, Kunitz, ShK), site-specific selection analysis of nucleotide sequences was performed using MEME and FEL. The branch-site test was conducted by aBSREL. The gene-wide episodic diversifying selection was performed using BUSTED.

To quantitatively evaluate the role of nematocyst proteins in cnidarian adaptation, orthologs in the genomes of eight cnidarians (*Acropora digitifera*, *H. vulgaris*, *Orbicella faveolata*, *Stylophora pistillata*, *Kudoa iwatai*, *T. kitauei*, *Exaiptasia diaphana*, *N. vectensis*) [35,93,94,95,96,97,98,99] were identified by OrthoMCL. We then classified these proteins into nematocyst proteins (NEMs) and proteins with no nematocyst-match (non-NEMs) by blasting against a protein repertory containing the same eight nematocyst proteomes as in comparative proteomics. We used the multiple alignments of the coding sequences from the eight cnidarians listed above to quantify adaptation across cnidarians using a method modified from Enard et al. [100]. Briefly, BUSTED was used as a threshold to determine whether genes were included in analyses. If the threshold for BUSTED *p*-value was set to 10^−x^, we only included in the quantification the adaptation of coding sequences with BUSTED *p*-value ≤ 10^−x^. For a certain BUSTED *p*-value, we assessed the amount of adaptation experienced by a particular protein (NEMs or non-NEMs) by estimating the average proportion of selected codons from the aBSREL test along all cnidarian branches. Then, the adaptation for whole NEMs (or non-NEMs) was quantified as the average proportions of selected codons in branches across NEMs (or the same number of randomly matched non-NEMs; see the permutation scheme described below). We then compared NEMs and non-NEMs for weak (BUSTED *p*-values ≤ 0.9) to increasingly strong (BUSTED *p*-values ≤ 10^−x^) evidence of adaptation. We calculated the excess of adaptation across cnidarians in NEMs, which is measured as the extra percentage of adaptation in NEMs compared to non-NEMs.

Non-NEMs were sampled by the permutation scheme. We used PAML to calculate dN and dS under M8 model [101] (Dataset S4). The average dN in NEMs was first calculated and noted as dN *_(NEM)_*. Then, we formed a range in which the average dN of sampled non-NEMs should fall. The infimum and supremum were defined as dN *_(inf)_* = 0.95 (αdN *_(NEM)_*) and dN *_(sup)_* = 1.05(αdN *_(NEM)_*). We then started to sample five non-NEMs randomly and continued until the average dN fell into [dN *_(inf)_*, dN *_(sup)_*]. The average dN of non-NEMs was kept within that interval. However, for every X non-NEM, the sampling was completely random. If the average dN of non-NEMs was not within [dN *_(inf)_*, dN *_(sup)_*], we continued to sample until it went back to our expected interval. In this way, the X in fact controls the variance of the sampling results, and the combination of α and X can be tested through a series of trial and error. To define α and X, we conducted 10^4^ random samplings of non-NEMs, and it was found that α = 0.95 and X = 3 yielded the closest dN/dS variance (0.001043401 in NEMs vs. 0.001075036 in non-NEMs, *p* = 0.7631) and slightly different dN/dS means (NEMs mean = 0.04989917 vs. non-NEMs mean dN/dS = 0.0551787, *p* = 0.0206). Further, Fisher’s exact test was used to determine whether there was adaptation enrichment in NEMs considering the whole cnidarian orthologs as a background.

## 3. Results

### 3.1. Comparative Proteomics

**Figure 1:**
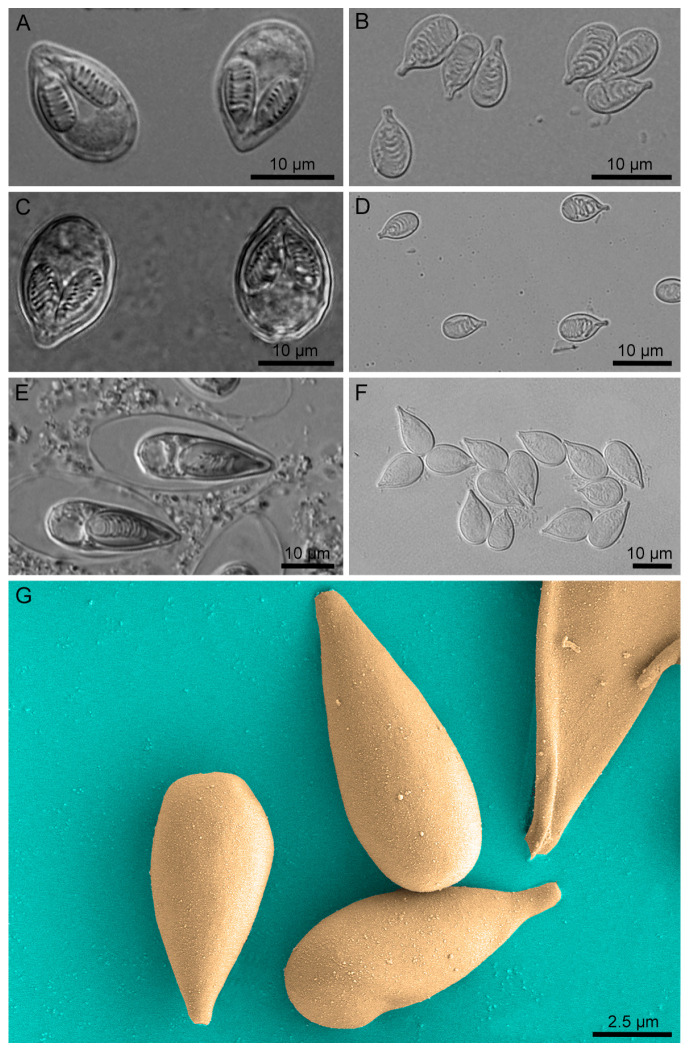
Light microscopy and scanning electron microscopy of myxozoan nematocysts. (**A**,**C**,**E**) Fresh and intact spores of *Myxobolus honghuensis*, *Myxobolus wulii*, and *Thelohanellus kitauei*. (**B**,**D**,**F**) Isolated nematocysts of *M. honghuensis*, *M. wulii*, and *T. kitauei*. (**G**) Scanning electron microscopy of the isolated *T. kitauei* nematocyst.

**Figure 2:**
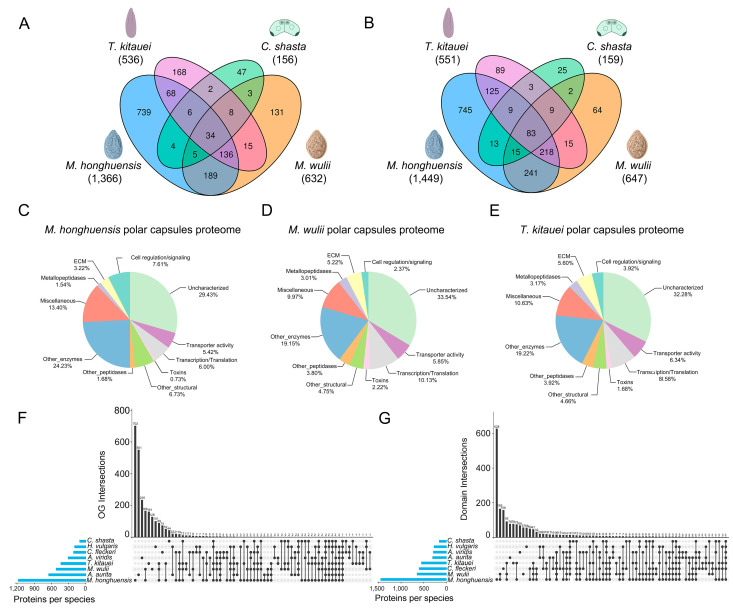
Comparative analysis of the newly obtained *Myxobolus honghuensis*, *Myxobolus wulii*, and *Thelohanellus kitauei* proteomes and published nematocyst proteomes for the myxozoan *Ceratonova shasta* and free-living cnidarians *Hydra vulgaris*, *Anemonia viridis*, *Aurelia aurita*, and *Chironex fleckeri*. Venn diagram showing the number of proteins (**A**) and InterPro domains (**B**) shared among the nematocyst proteomes of *M. honghuensis*, *M. wulii*, *T. kitauei*, and *C. shasta*. (**C**–**E**) Distribution of functional nematocyst proteomes. Functional protein classes of the *M. honghuensis*, *M. wulii*, and *T. kitauei* nematocyst proteomes. Upset diagram showing the number of proteins (**F**) and InterPro domains (**G**) shared among the eight cnidarian nematocyst proteomes.

Three new nematocyst proteomes were obtained for *M. honghuensis* (1366 NEMs), *M. wulii* (632 NEMs), and *T. kitauei* (536 NEMs) of the family Myxobolidae (Figure 1, Dataset S1). Comparative proteomics revealed that only 49, 50, and 50 NEMs were shared between these myxobolids and *C. shasta* (family Ceratomyxidae, 114 NEMs [32]) (Figure 2A). Within the myxobolids, only 244 and 193 NEMs were shared between *T. kitauei* and *M. honghuensis* and between *T. kitauei* and *M. wulii*, respectively. Even between the closely related *M. honghuensis* and *M. wulii* (congeners), only 364 NEMs were shared, constituting 30.8% and 69.9% of the total NEMs. Thirty-four orthologs were conserved among myxozoan NEMs, including gamma-glutamyl transpeptidase, nematoblast-specific nb012a, and rootletin (Dataset S5). In terms of quantity, nematogalectins and nematoblast-specific nb012a accounted for a relatively higher proportion of the total NEMs. However, the abundance pattern of the 34 NEMs was not significantly associated with species phylogenetic relationships.

Only 120, 109, and 104 domains were shared between the myxobolids and *C. shasta* (Figure 2B). Within the myxobolids, 435 and 325 domains were shared between *T. kitauei* and *M. honghuensis* and between *T. kitauei* and *M. wulii*. A total of 557 domains were shared between *M. honghuensis* and *M. wulii*, constituting 38.4% and 86.1% of the total domains. Eighty-three domains were conserved among myxozoan NEMs. Contrary to the limited number of shared domains, all myxozoan NEMs had highly similar functional distributions. The proteomes were characterized by enzymes, structural proteins, and novel sequences. Many enzymes were part of the venom proteome, and structural proteins were composed mainly of sequences with ECM motifs (Figure 2C–E). A global comparison including free-living and parasitic cnidarians showed that the species-specific components ranked the highest for both OG (orthologous groups) and domain intersections (Figure 2F,G).

**Figure 3:**
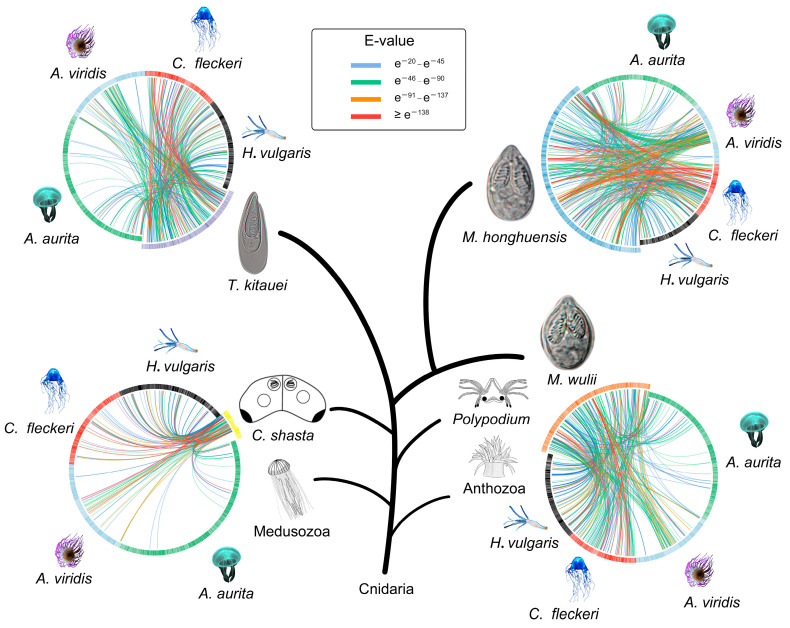
Circos representation of the sequence similarities of identified nematocyst proteins from the four myxozoan models with published free-living cnidarian nematocyst proteomes (−*e* value 1 × 10^−20^). Colored lines represent similarity scores, with the corresponding E-values denoted in the figure.

We next focused on the inter-group comparison of NEMs between myxozoans and free-living cnidarians. Estimates of sequence similarity were inconsistent with phylogenetic relationships (Figure 3). For example, all myxozoans had more common proteins and higher amino acid sequence similarity to those of the sea anemone *A. viridis* than to those of the moon jellyfish *A. aurita*. Likewise, there were as many as 201 NEM matches between *M. honghuensis* and *A. viridis*, nearly twice as many matches obtained for *M. honghuensis* vs. *H. vulgaris*.

### 3.2. Myxozoan Venom Analysis

**Figure 4:**
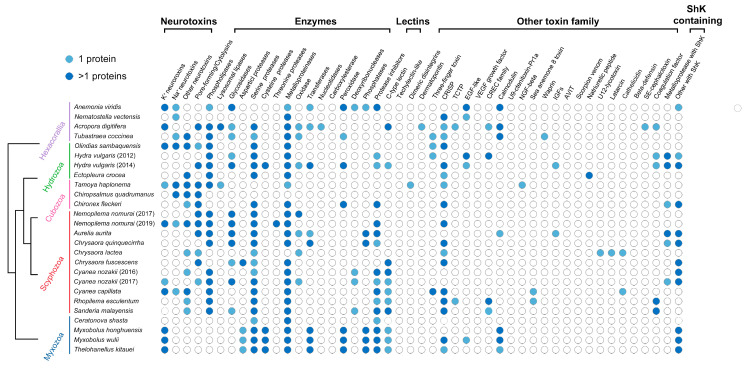
Presence of toxin families in cnidarian proteomes. A schematic tree of Cnidaria is shown on the left. Circles indicate the presence of a toxin family in a species, light blue indicates that only one protein was identified in the nematocyst proteome, and dark blue indicates that more than one protein was identified in the nematocyst proteome.

In total, 67, 74, and 67 putative toxins were identified in the *M. honghuensis*, *M. wulii*, and *T. kitauei* proteomes, respectively (Dataset S2). According to their predicted biological functions, these toxins were classified into five categories (Figure 4). The proportional distributions of these toxin families were similar among the three species (Dataset S2). Peptidases were the most diverse putative toxins, including metalloproteinases, cysteine proteases, serine proteases, and aspartic peptidases. The metalloproteinases were alanine aminopeptidases, astacins, ADAMs, carboxypeptidases, and thimet oligopeptidases. The relative distributions of the other enzymes in the three species were also similar and, in particular, were dominated by transferases, phosphatases, and peroxidases. In addition, other toxins, such as neurotoxins (ShK-like and Kunitz-type), protease inhibitors, C-type lectins, and CRiSPs, were identified in the NEMs of the three myxobolids.

Overall, the myxozoan toxin family was under negative selection (ω < 1) (Dataset S6). However, using the branch-site unrestricted statistical test for episodic diversification (BUSTED), we found evidence for episodic gene-wide positive selection across the myxozoan astacin-like toxin family (*p* = 0.007). This indicates that at least one site on at least one branch of the myxozoan-dominated astacin-like toxin subclade underwent positive selection based on a comparison with the free-living cnidarian branches and genes. Using adaptive branch-site random effects likelihood (aBSREL), we also found evidence for episodic positive selection on the *M. honghuensis* branch in the C-type lectin phylogeny.

### 3.3. NEM Core Set

The nematocyst core set (NEMs shared by all eight cnidarians evaluated in the present study) included five proteins, most of which have housekeeping functions (Table 1). Elongation factor 1 alpha (EF1α) is a key component of protein biosynthesis [102]. Histones (H2A and H2B) are the chief protein components of chromatin [103]. Heat shock proteins are important molecular chaperones [104]. Nematogalectin is a major structural component of the nematocyst tubule [105]. We then performed database searches and phylogenetic analyses of these proteins to determine the variant types. The EF1α and HSP (heat shock protein) sequences were identified as EF1α-1 and heat shock cognate 71 kDa (Hsc70) based on sequence comparisons. The histones were assigned to canonical H2A and canonical H2B type using HistoneDB2.0 [106]. The nematogalectin was identified as the ancient nematogalectin-related type by a gene family analysis of cnidarian nematogalectins (Figure S4).

### 3.4. Cnidarian Organismal and Nematocyst Phylogenetic Analyses

**Figure 5:**
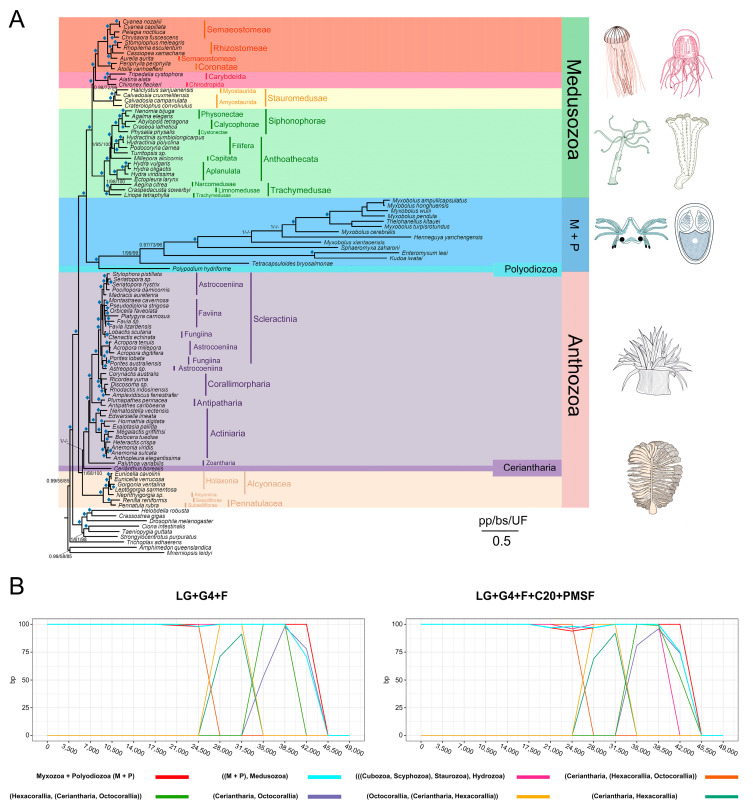
(**A**) Tree of Cnidaria. The topology was inferred from the concatenated amino acid sequence (main matrix 4_103tx_146og, 103 species, 51,598 positions) using PhyloBayes. Values at nodes are posterior probabilities (pp) under CAT + GTR + Γ4, nonparametric ML bootstrap (bp) values obtained from 100 ML replicates using the PROTGAMMAAUTO model implemented in RAxML, and ultrafast bootstrap (UF) values from IQ-TREE under the LG + Γ4 + FMIX (empirical, C20) + PMSF model. Diamond marks on branches correspond to 1.0 Bayesian pp and >95% bp and UF. Dashes represent the alternative maximum likelihood topology. (**B**) Fast-evolving site removal analysis. Sites were sorted based on their rates of evolution estimated under the LG + Γ4 + F model and were removed from the data set according to rates (from highest to lowest). In each step, 3500 of the fastest evolving sites were removed. The bootstrap values for each bipartition of interest are plotted under LG + Γ4 + C20 + F + PMSF.

Our initial cnidarian organismal set consisted of 105 taxa, 146 orthologous groups, 51,598 amino acid positions, and 24.8% missing data (Figure S5). Both Bayesian analyses using the CAT model and a maximum likelihood (ML) analysis using the PMSF model strongly recovered the following relationship: (((Myxozoa + *Polypodium*) + Medusozoa) + Anthozoa). However, the myxozoan *H. yanchengensis* was grouped within Bilateria (Figure S6) even after sequence bias correction (Figures S7–S9) and rogue species deletion (Figures S10–S12). We then applied decontamination methods to *Henneguya* according to Kayal et al. [107], and the resulting matrix robustly supported the assignment of *H. yanchengensis* to the myxozoans (Figure S13). We ultimately obtained a well-supported topology with good resolution of relationships among all major lineages of cnidarians (Figure 5A), consistent with recently proposed relationships within Cnidaria [107].

Approximately unbiased (AU) tests were performed to compare alternative topologies proposed in previous studies or obtained in our analysis (Figure S14). Most alternative topologies for Myxozoa + *Polypodium* were significantly rejected (Table 2). To further examine these conflicts, we estimated the rate of evolution at sites in the supermatrix of 146 proteins from 105 taxa and removed sites in a stepwise manner according to the rate of evolution, plotting bootstrap values for nodes of interest in the tree per site deletion step under the models LG + F + Γ4 and LG + C20 + F + Γ4 + PMSF estimated using IQ-Tree (Figure 5B). With the progressive removal of the fastest evolving sites, the topologies estimated by both the site-homogeneous and site-heterogeneous model and their support values were unaffected until well over half of the data set was removed. Overall, the conventional topology for the main lineages of cnidarians and class-level relationships within Medusozoa were consistently and robustly supported.

**Figure 6:**
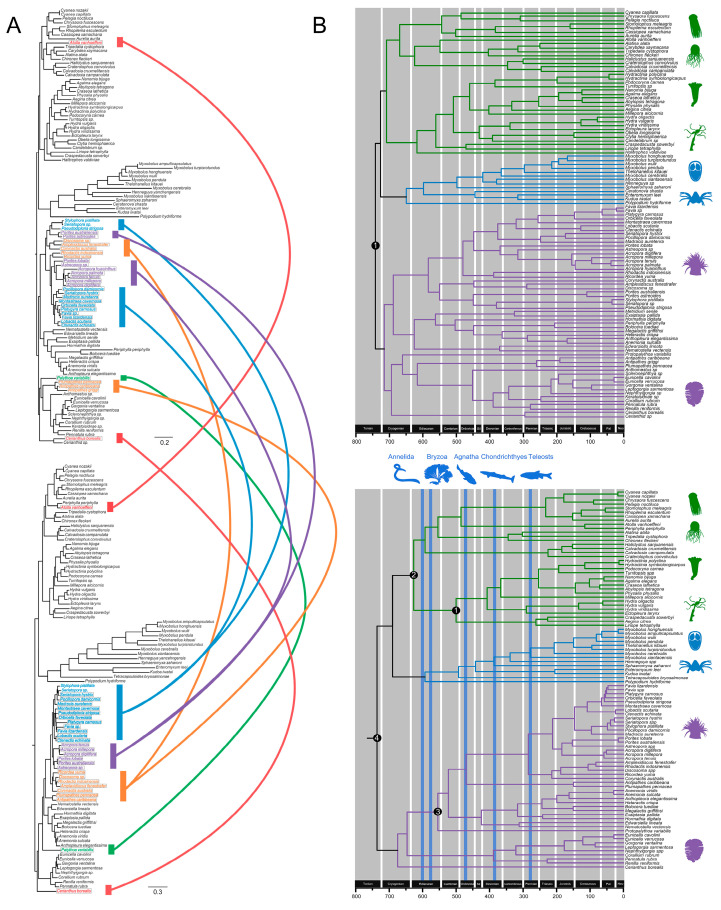
(**A**) Congruence between the nematocyst core protein tree (upper panel) and organismal tree (lower panel). Nematocyst protein tree based on the concatenated protein alignment of five nematocyst core proteins. Species tree based on the concatenated protein alignment of 146 single-copy proteins. Cnidarian groups are shaded to highlight incongruencies resulting from gene transfer events. (**B**) Chronogram of nematocyst (upper panel) and cnidaria (lower panel) evolution inferred using r8s. We used the phylogeny resulting from concatenations of five and 146 genes in our age estimation. Calibration points for nematocyst chronogram were as follows: (1) Cnidaria max-age (~741 Mya) is marked with solid circles. Calibration points for the Cnidaria chronogram: (1) origin of Hydrozoa (500 Mya), (2) Medusozoa minimum age (~570 Mya), (3) Hexacorallia min age (~540 Mya), and (4) Cnidaria max-age (~741 Mya) are marked with four solid circles. Neo = Neogene, Pal = Paleogene, Sil = Silurian.

The nematocyst tree successfully recovered the higher-level phylogeny of Cnidaria: (((Myxozoa + *Polypodium*) + Medusozoa) + Anthozoa) (Figure 6A). We also recovered the two sister clades, Octocorallia and Hexacorallia, in Anthozoa and a monophyletic Medusozoa with the well-accepted topology of (((Cubozoa + Scyphozoa) + Staurozoa) + Hydrozoa). However, the nematocyst phylogenetic reconstruction was inconsistent with the lower-level taxonomy of Cnidaria (i.e., at the genus or species levels).

### 3.5. Major Events in Nematocyst Evolution

In the NEM core set, evolutionary events before the diversification of the main lineage only involved the gain of genes encoding nematogalectin/minicollagen and co-option of the housekeeping histones H2A, H2B, EF1α, and Hsc70. By comparing the phylogeny of cnidarians (Figure 5, Table 2) with that of nematocyst core proteins (Figure 6A), we examined the evolution of the core set after diversification of the main lineage and found several inconsistencies in the placement of Cerianthidae, Antipatharia, and *Palythoa variabilis*. This most likely resulted from the transfer of the entire nematocyst gene complexes between Anthozoa lineages after their separation from other major cnidarian groups. In addition to gene transfer, post-expansion evolutionary events in the putative core set included the expansion of nematogalectin and minicollagen (if counted) by duplication and divergence.

By contrast, the non-core NEM set showed massive gene recruitment, gene duplication, and gene decay events. Some toxins, such as the Kunitz-type family, were recruited independently into Anthozoa/Scyphozoa/Myxobolidae; the Kunitz-type family was duplicated in Myxobolidae (Figure S15). Actinoporins were gained at the base of cnidarians and subsequently lost in myxozoans. All venom proteins were lost in *Ceratonova* (Figure 4). We also observed the extensive domain shuffling of the ShK toxin (Figure S16) and domain recruitment in C-type lectin (Figure S17) and metalloproteinases (Figure S18).

### 3.6. Divergence Time Estimates

All divergence time estimates are summarized in Table 3. Nematocyst evolution was always one step ahead of species evolution, with a gap of 33–55 million years (Figure 6B). The last common ancestor of cnidarians occurred during the Tonian period, approximately 736 Mya. Medusozoa originated in 626.6 Mya. We estimated that the most recent common ancestor (MRCA) of all myxozoans dates back to the late Cambrian (492.6 Mya).

### 3.7. NEM and Non-NEM Selection and Adaptation Analyses

**Figure 7:**
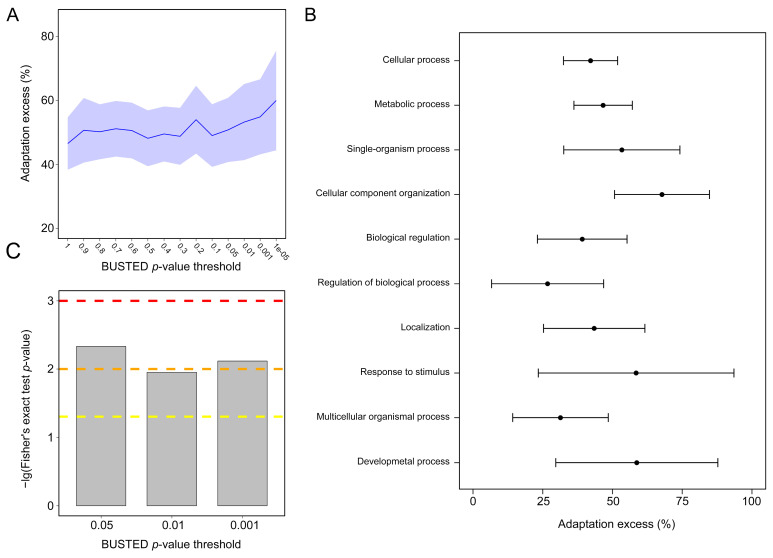
The significant contribution of the nematocyst to cnidarian protein adaptations. (**A**) Excess of adaptation across cnidarians in nematocyst proteins (NEMs). The excess of adaptation is measured as the excess percentage of adaptation in NEMs compared to non-NEMs. The shaded part represents the 95% confidence interval for the excess of adaptation in all NEMs. (**B**) Excess of adaptation within the top 10 high-level GO processes with the most NEMs. Excess is shown for BUSTED *p* ≤ 0.5. (**C**) The adaptation enrichment in NEMs detected by Fisher’s exact test. Dashed yellow curve, orange curve, and red curve: Fisher’s exact test *p*-value thresholds of 0.05, 0.01, and 0.001.

All NEMs showed approximately 50% more adaptive amino acid changes, on average, compared to estimates in non-NEMs. NEMs with the strongest evidence of adaptation (BUSTED *p*-values < 10^−5^) had up to 60% excess of adaptive substitutions (Figure 7A, permutation test *p* < 2.2 × 10^−16^, 10^7^ iterations). These NEMs were enriched for 20 GO (Gene Ontology) functions, including eight GO categories associated with more than 50 NEMs. Among the top 10 GO categories, seven were related to NEMs with a 40% or greater excess of adaptation (permutation test *p* < 0.05 in all cases). GO terms associated with a strong excess of adaptation included cellular processes, such as metabolism, cellular component organization or biogenesis, and biological regulation, as well as supracellular processes related to development (Figure 7B). Moreover, Fisher’s exact test of NEMs against background proteins showed that selective pressure was significantly enriched in NEMs (*p* = 0.004658, 0.01117, and 0.007638 at the 0.05, 0.01, and 0.001 levels; Figure 7C).

## 4. Discussion

### 4.1. Nematocyst Heterogeneity and Organelle–Organism Discordance Support Extensive Species-Specific Adaptation

Our interspecific comparisons strongly support extensive species-specific adaptations in nematocyst evolution. Only 34 orthologs in nematocyst proteomes were shared among myxozoans. This heterogeneity was consistent with previous results for free-living cnidarians [21]. Comparing myxozoan data to data for free-living cnidarians, only four of the 34 orthologs were myxozoan-specific, and this number might be reduced in the future as sampling is expanded. Our results suggest that a myxozoan nematocyst “molecular toolbox” was lacking in the common ancestor and was potentially conserved among myxozoans for parasitism.

Despite the considerable differences in NEMs among cnidarians, there were a number of shared domains. Among myxozoans, the functional modules that make up the proteomes were highly similar. These results suggest that the composition and functional heterogeneity of the nematocyst resulted from extensive species-specific adaptation, a process focused more on functions than on specific protein types [21].

The discordance between proteomic similarity and phylogenetic relationships may also be explained by species-specific adaptations. Under changing conditions, the whole nematocyst proteome might have undergone rapid evolutionary changes. Thus, proteomic alterations might be inconsistent with the species phylogeny, especially for fast-evolving lineages. This prediction was tested by mapping proteomic results to the species phylogeny. Myxozoans had NEMs that were similar to those of sea anemones, inconsistent with the expectation that myxozoans are phylogenetically more closely related to Medusozoa than to Anthozoa [21], suggesting that rapidly evolving myxozoans are armed with nematocysts for parasitism. To investigate whether quantitative information from proteomics data would provide insights into species phylogenetic relationships, we compared the relative iBAQ values of 34 shared NEMs among three myxozoans. The abundance patterns of the 34 NEMs were found not to be significantly associated with phylogenetic relationships. However, our results may not reflect the true composition of nematocysts, as different proteins may perform differentially during digestion [21]. Thus, the relationship between the nematocyst protein abundance pattern and the phylogeny is still wide open for discussion, and more quantitative proteomics, including stable isotope labels and optimization of the dissolution method, are needed to quantify the nematocyst composition. Collectively, our results demonstrate that the nematocyst is highly diverse at the proteomic level. The striking interspecific heterogeneity in nematocyst proteome composition may be related to rapid diversification, highlighting its potentially significant role in adaptive evolution.

### 4.2. Dynamic Evolution of Myxozoan Venom and Episodic Positive Selection

Venom stored in nematocysts may be under selection, independent of other nematocyst core/structural proteins. Therefore, the evolution and selection of toxins warrant an independent investigation. We present evidence for the expression and translation of venom toxin homologs in myxobolid nematocysts, consistent with previous proteomic studies of myxospores [21]. Putative toxins are absent from *C. shasta* [32], suggesting that the composition of venom varies greatly among myxozoan families. The apparent loss of venom toxins in nematocysts of *C. shasta* could be attributed to a structural adaptation that facilitates host attachment rather than toxin delivery [40]. Despite the significant sequence differences, the functional modules of toxins were similar among the three myxobolids, exhibiting a similar evolutionary trend to that of other non-toxic NEMs. As observed in the comparative analysis of all NEMs, the Myxobolidae NEMs showed a unique toxin distribution pattern that was more similar to that anthozoans than to medusozoans, including abundant putative neurotoxins and a lack of cytolysins [108,109,110]. Our results are inconsistent with the hypothesis that the toxin gene distribution is correlated with species relatedness [108], suggesting that toxin families are not necessarily restricted to certain taxonomic groups but are common throughout cnidarian venom evolution [27].

Animal venoms are theorized to evolve under positive Darwinian selection in an arms race scenario [21]. Previous work has shown that genes encoding toxins in cnidarians experience stronger purifying selection than those of other venomous animals, possibly due to the functional role of these proteins in nematocyst development [21,111,112]. In myxozoans, despite substantial purifying selection across toxin sequences, a subset of genes (i.e., sites) along a subset of lineages showed evidence for positive selection, referred to as episodic positive selection [112]. We speculate that due to the simple morphology, myxozoan venoms are less constrained by nematocyst development. Thus, genomic constraints imposed on venom evolution are partially relaxed, leading to the intensification of selection on myxozoan toxins. Furthermore, this discrete positive selection may correspond with increased potency and/or specificity of a particular toxin or species. Overall, these patterns suggest that myxozoan venoms were subjected to various selective pressures, including wide-spread purifying selection and a few instances of gene-specific or lineage-specific episodic positive selection.

### 4.3. Defining the NEM Core Set

The identification of the core protein set is important for analyses of nematocyst evolution. In accordance with Rachamin et al. [21], we validated the relatively small set of core NEMs, most of which are housekeeping proteins. They include two histones, HSP, EF1α, and nematogalectin. Only nematogalectin is a nematocyst-specific component. Thus, it is possible that these non-nematocyst-specific components are cellular contaminants that can be explained by density gradient centrifugation, leading to the co-elution of nematocyst components with a small fraction of nuclei. However, we think that this explanation is unlikely for several reasons. (1) The purity of nematocysts was verified by light and electron microscopy. (2) The house-keeping proteins have also been reported in previous studies of free-living cnidarians [21,24] and are thought to have non-canonical functions, such as the maintenance of venom homeostasis (HSP), shaping the microtubule scaffold (EF1α), and even acting as venom (histones). (3) Histones have also been recovered from the clean nematocysts of *C. shasta* extracted by a dielectrophoresis-based microfluidic device. (4) The fraction of these house-keeping proteins in the complete proteome was high, as estimated by iBAQ values. Thus, their presence cannot be explained by chance alone. (5) The house-keeping proteins may have been co-opted by the ancestral nematocyst, suggesting that the core proteins are not necessarily nematocyst-specific. This is supported by the observation that all the core NEMs were the canonical type, in accordance with their ancient origin.

Next, conspicuously missing from our results were the minicollagens detected by Balasubramanian et al. [18], one of the most comprehensive datasets generated to date. This could reflect our isolation process or an insufficient protein solubilization protocol for the resolution of most major structural components, such as minicollagens. However, we do not think the absence of minicollagens is the result of insufficient nematocyst solubilization for three reasons. (1) Minicollagens formed insoluble polymers in mature wall structures and were difficult to dissolve in SDS before analysis, as observed by Rachamin et al. [21], and may reflect differences in extraction methods. (2) We successfully resolved major structural components, such as nematogalectins and ECM-related proteins, constituting about one-third of the *Hydra* nematocyst proteome [18]. (3) The proportion of structural proteins in our NEMs was lower than that in *Hydra* but comparable to that reported for the myxozoan *C. shasta* [32]. Thus, the reduction in structural components in our NEMs might be explained by lineage-specific characteristics, rather than insufficient solubilization. We performed a further proteomic database search using the oxidation of proline and still did not detect minicollagens (Dataset S7). Hence, the lack of minicollagen detection is not due to failure of matching collagenous peptides with proline hydroxylation. Despite the important role of minicollagen in nematocysts, it was not included in the current core set; it shows a complex evolutionary history and the ancestral sequence is unclear [113,114]. Therefore, the task of elucidating the evolution of the nematocyst may rest on establishing how the above core set evolved.

### 4.4. Decentralized Evolutionary Pattern of Nematocysts

With the aid of a robust cnidarian phylogeny and a nematocyst tree based on the core set, the evolution of the core/non-core set before and after the major lineage expansion was examined. We found a pattern of “decentralized modification”, in which the putative core set underwent few modifications before and after appearing in the shared ancestor of cnidarians, and most evolutionary events were found in the non-core set. For example, the evolutionary processes involving the core set only included a few innovations, co-option events, expansion of nematogalectin/minicollagen, and frequent gene transfer events in anthozoans (Figure 6A), the latter of which might be related to the frequent symbiosis between anthozoans and dinoflagellates algae [115]. The non-core set, however, showed massive gene recruitment, gene duplication, and gene decay events. The above evolutionary pattern is distinct from that of other organelles or complex systems. e.g., bacterial flagella [116], bivalve shell matrix [117] or bacterial non-flagellar T3SS (NF-T3SS) [118].

The most plausible explanation for the decentralized modification is that the rise of a nematocyst prototype was followed by the rapid diversification of cnidarians and, therefore, there was a relatively short period for evolutionary events to occur [119] before the rapid expansion of nematocysts into various lineages. Subsequently, strong selective pressure led to active lineage-specific modifications and finally contributed to the high heterogeneity of nematocysts. These results provide reasonable support for the key role of the nematocyst in cnidarian evolution.

### 4.5. Macroevolutionary Lag between the Nematocyst Core Set and Cnidarian Diversification

A prerequisite for the nematocyst to be an adaptive driver is that the ancestral nematocyst arose before the main cnidarian lineages diverged. This was tested by comparing the timescale of the nematocyst ancient core complex and organismal evolution under the same chronogram framework. The nematocyst consistently predicated steps in cnidarian evolution with a stride length of 33–55 My. The last common ancestor of cnidarians occurred during the Tonian period, approximately 736 Mya, within the range of 389–1035 Mya [120], earlier than 561–626 Mya [121], but later than the estimates reported by Holzer et al. [5] (723–848 Mya), Park et al. [90] (686–819 Mya), and Waggoner and Collins [122] (700–1200 Mya). Medusozoa was estimated to have originated 626.6 Mya, which is earlier than estimate of Park et al. [90] (571–670 Mya) but later than that of Waggoner and Collins [122] (640–940 Mya). In shallower nodes, such as the origin of cnidarian classes or families, our divergence time estimates are considerably younger than those of Holzer et al. [5]. We estimated that the most recent common ancestor (MRCA) of all myxozoans arose in the late Cambrian (492.6 Mya), later than the Ediacaran, 588 Ma (540–642 Mya), reported by Holzer et al. [5]. These results suggest that nematocysts achieved substantial complexity early in the evolution of these groups and have the potential to be a driving evolutionary force.

### 4.6. Frequent Adaptation in NEMs across Cnidarians

Despite evidence for the essential role of the nematocyst in cnidarian adaptive evolution, the extent to which the trait contributed to the evolution of the lineage remains unclear. For quantitative analysis, we detected episodes of adaptive evolution along each of the eight branches on the cnidarian tree using the adaptive branch-site REL test (aBSREL test) [123] and the BUSTED test [124]. A permutation test showed that adaptation has been much more common in NEMs than in non-NEMs across cnidarians. The strongest excess of adaptation in NEMs reached 60%, and these were related to 20 GO functions. The excess of adaptation in NEMs therefore involves wide functions. Interestingly, some of these functions are consistent with the functions of recently diverged nematocysts, such as response to stimuli and organelle localization. Moreover, a selective pressure enrichment analysis of the NEMs against conserved background proteins indicated that selective pressure on background proteins was significantly enriched in the NEMs, consistent with the idea that selection in cnidarians disproportionately affects the nematocyst. These results suggest that the nematocyst plays a predominant role in adaptive evolution in cnidarians via pleiotropic effects on diverse biological functions.

### 4.7. Future Perspectives

Our analysis benefits from the integration of decades of omics data for cnidarians. However, there are still potential sources of bias that should be addressed in future research. Although our NEM core set is in accordance with previous studies, it may change over time as a result of increased taxon sampling and the optimization of nematocyst solubilization methods. Furthermore, additional genome- and transcriptome-wide searches of cnidarian minicollagens are needed to better clarify the evolutionary trajectories of these proteins [114], which can be integrated into the core set and further improve our understanding of nematocyst evolution.

## 5. Conclusions

Starting with a top-down analysis of a phenotype of interest, we observed a strong increase in the rate of adaptive evolution in the nematocyst by a holistic consideration of the proteomic composition, patterns of evolution, chronograms, and quantification of adaptive evolution. The nematocyst likely increases evolvability, enabling adaptation to changing selection regimes and thereby contributing to the striking diversity of cnidarians. The unique role of nematocysts provides an important addition to the catalog of mechanisms underlying adaptive success in the lineage and establishes a promising system for investigating the ecological and evolutionary implications of phenotypic novelties in non-model organisms. Our results emphasize the importance of quantitative analyses in the role of novelties in evolution and diversification, providing a basis for reassessing the evolutionary history of many established models, with the potential to substantially improve our understanding of both the mechanistic basis of novel traits and evolutionary processes. The present study and other research on cnidarian structures also play an important role as simple models for basic theories of structural development–beginning with the pioneering work of Alan Turing [125,126,127,128,129].

## Figures and Tables

**Table 1:** Putative nematocyst core set that was conserved through cnidarians.

Protein	*M. honghuensis* ID	*M. wulii* ID	*T. kitauei* ID
Elongation factor 1 alpha 1	75931_MH_MC	23790_WL_MC	165085_TK_MC
Canonical histone H2A	32284_MH_MC	49939_WL_MC	80662_TK_MC
Canonical histone H2B	28864_MH_MC	51458_WL_MC	62843_TK_MC
Heat shock cognate 71 kDa (Hsc70)	8642_MH_MC	159445_WL_MC	85197_TK_MC
Nematogalectin-related	75931_MH_MC	23790_WL_MC	165085_TK_MC

**Table 2:** Approximately unbiased test of the cnidarian phylogeny. Each tree was loosely constrained with the hypothetical groupings and optimized under LG + Γ4 + FMIX (empirical, C20) in IQ-tree using the 146-gene (51,598 sites) data set. The optimized trees were compared using the approximately unbiased test with 10,000 RELL bootstrap replicates. Maximum log-likelihoods of each constraint and their differences from the optimal ML tree are listed. The hypotheses within the 95% confidence interval that could not be rejected are where *p* ≤ 0.05. POLYPODIOZOA, *Polypodium hydriforme*.

Matrix_Name	Constraint_Tree_Iqtree	Log L	Δlog L	*p*-Value AU	Bootstrap RELL
4_103tx_146og	Best_iqtree_ML	−2,303,638.671	0.000	0.6220	0.4661
	Best_Bayes	−2,303,688.375	49.704	0.1477	0.1261
	[Polypodiozoa + Hydrozoa]	−2,304,312.129	673.458	0.0001	0.0000
	[Polypodiozoa + Aegina_citrea]	−2,305,903.793	2265.122	0.0018	0.0000
	[[[Myxo + Polypodiozoa] + Anthozoa] + Medusozoa]	−2,304,295.467	656.796	0.0004	0.0000
	[[Medusozoa + Octocorallia] + Hexacorallia]	−2,305,100.274	1461.603	0.0025	0.0000
	[[[Ceriantharia+ Hexacorallia] + Octocorallia] + Medusozoa]	−2,303,639.715	1.044	0.5813	0.4078
	[[[Cubozoa+ Scyphozoa] + Hydrozoa] + Staurozoa]	−2,303,758.459	119.787	0.0001	0.0000
	[[Staurozoa + Cubozoa] + [Hydrozoa + Scyphozoa]]	−2,304,131.216	492.544	0.0000	0.0000

**Table 3:** Comparison of divergence times for major nodes shared across eight studies. Numbers in the parentheses represent the median times and 95% HPD interval (Mya). “-” = Not available. “M + P” means Myxozoa + Polypodiozoa; “Coral” means Corallimorpharia.

	Present Study	Holzer et al. 2018	Kodádková et al. 2015	Park et al. 2012	Waggoner & Collins 2004	Cartwright & Collins 2007	Medina et al. 2006	Peterson et al. 2004
Markers	146 single-copy nuclear orthologs	6 nuclear protein-coding genes	18S rRNA gene	13 mitochondrial protein-coding genes	18S rRNA gene	18S and 28S rRNA genes	13 mitochondrial protein-coding genes	7 nuclear protein-coding genes
Node age								
**Deep nodes**								
Cnidaria	736.2	786 (723–848)	-	741 (686–819)	875 (700–1200)	711.7 (389, 1035)	-	595 (561–626)
“M + P”	592.9	725 (667–779)	-	-	-	-	-	-
Medusozoa	626.6	-	-	607 (571–670)	790 (640–940)	-	-	-
“M +P” + Medusozoa	689.6	-	-	-	-	-	-	-
**Shallow nodes**								
*P. hydriforme*	592.9	651 (601–700)	-	-	-	-	-	-
Myxozoa	492.6	588 (540–642)	530 (457–603)	-	-	-	-	-
Cubozoa	344.5	-	-	-	-	-	-	-
Scyphozoa	485.3	-	-	-	-	-	-	-
Cubozoa + Scyphozoa	554.2	-	-	-	-	537.5 (191–943)	-	-
Staurozoa	225.5	-	-	-	-	-	-	-
Octocorallia	388.5	-	-	292 (173, 421)	-	-	-	-
Hexacorallia	554.4	-	-	601 (544, 684)	-	517.5	-	-
Scleractinia + Cora	284.3	-	-	276 (235–323)	-	-	264 (240–280)	-
Cora	214.2	-	-	206 (158–253)	-	-	121 (110–132)	-

## Data Availability

Data generated and analyzed during this study are included in the published article, its additional files, and publicly available repositories. The mass spectrometry proteomics data have been deposited to the ProteomeXchange Consortium via the PRIDE [130] partner repository with the dataset identifier PXD030136. The raw reads of *M. honghuensis* transcriptome sequencing and Illumina genome sequencing have been deposited in the NCBI Short Read Archive (SRA) with the Bioproject accession numbers PRJNA779260 and PRJNA778632. The raw reads of *M. wulii* transcriptome sequencing and Illumina genome sequencing have been deposited in the NCBI SRA with the project accession number PRJNA786090 and PRJNA786089. The raw reads of other myxozoan transcriptome sequencing were also deposited under the Bioproject accession numbers in GenBank: *M. ampullicapsulatus* (PRJNA786097), *M. turpisrotundus* (PRJNA786098), *M. xiantaoensis* (PRJNA786274), *H. yanchengensis* (PRJNA786290), *T. kitauei* (PRJNA786278, Illumina Hiseq 2500), and *T. kitauei* (PRJNA786276, Illumina Hiseq 4000). The phylogenetic trees, alignments, and data of fast-evolving sites removal analysis have been deposited in the Harvard Dataverse: https://doi.org/10.7910/DVN/DKUG4Y (accessed date: 16 December 2021).

## Supplementary Materials

The following are available online at https://www.mdpi.com/article/10.3390/biology11010091/s1, Figure S1: Protein quantitation of the isolated myxozoan nematocysts, Figure S3: Flowchart of phylogenomic data analysis, Figure S4: Cnidarian nematogalectin ML tree, Figure S5: Visualization of the supermatrix for final phylogenomic constructions, Figure S6: Cnidarian tree inferred by IQ-TREE (105 taxa and 51,598 amino acid sites), Figure S7: Heat map of the taxon-specific long-branch scores for the cnidarian tree (105 taxa and 51,598 amino acid sites), Figure S8: Density plots of different gene-specific long-branch indices for the 146 genes of the cnidarian tree, Figure S9: Density plots of different gene specific saturation indices for the 146 genes of the cnidarian tree, Figure S10: Cnidarian tree inferred by RAxML (105 taxa and 51,598 amino acid sites), Figure S11: Cnidarian tree inferred by IQ-TREE (103 taxa and 42,573 amino acid sites), Figure S12: Cnidarian tree inferred by IQ-TREE (102 taxa and 42,573 amino acid sites), Figure S13: Cnidarian tree inferred by IQ-TREE (103 taxa and 51,598 amino acid sites), Figure S14: Alternative topologies among major lineages of cnidarians, Figure S15: Bayesian analysis of the cnidarian type-2 K+ channel toxins, Figure S16: Bayesian analysis of the cnidarian ShK-like toxin domains, Figure S17. Bayesian analysis of the cnidarian C-type-lectin domains, Figure S18: Bayesian analysis of the cnidarian astacin (M12A metalloproteinases), Table S1: Sequencing and assembly statistics for genomes and transcriptomes in this study, Dataset S1: List of nematocyst proteins identified from three myxozoans (each with three replicates), Dataset S2: Overview of the toxin diversity of representative cnidarian species, Dataset S3: Taxa included in the phylogenomic study, Dataset S4: dN and dS of all cnidarian conserved orthologs calculated by PAML under M8 model, Dataset S5: List of the 34 orthologs that are conserved among four myxozoan nematocyst proteins, Dataset S6: Selection analyses of the myxozoan toxin families, Dataset S7: List of nematocyst proteins identified from three myxozoans (each with three replicates and oxidation of proline parameter added).

## References

[1] Cartwright P., Halgedahl S.L., Hendricks J.R., Jarrard R.D., Marques A.C., Collins A.G., Lieberman B.S. (2007). Exceptionally Preserved Jellyfishes from the Middle Cambrian. PLoS ONE.

[2] Bhattacharya D., Agrawal S., Aranda M., Baumgarten S., Belcaid M., Drake J.L., Erwin D., Foret S., Gates R.D., Gruber D.F. (2016). Comparative Genomics Explains the Evolutionary Success of Reef-Forming Corals. Elife.

[3] Atkinson S.D., Bartholomew J.L., Lotan T. (2018). Myxozoans: Ancient Metazoan Parasites Find a Home in Phylum Cnidaria. Zoology.

[4] Hallett S.L., Hartigan A., Atkinson S.D., Okamura B., Gruhl A., Bartholomew J.L. (2015). Myxozoans on the Move: Dispersal Modes, Exotic Species and Emerging Diseases. Myxozoan Evolution, Ecology and Development.

[5] Holzer A.S., Bartošová-Sojková P., Born-Torrijos A., Lövy A., Hartigan A., Fiala I. (2018). The Joint Evolution of the Myxozoa and Their Alternate Hosts: A Cnidarian Recipe for Success and Vast Biodiversity. Mol. Ecol..

[6] Lotan A., Fishman L., Loya Y., Zlotkin E. (1995). Delivery of a Nematocyst Toxin. Nature.

[7] Beckmann A., Özbek S. (2012). The Nematocyst: A Molecular Map of the Cnidarian Stinging Organelle. Int. J. Dev. Biol..

[8] Young G.A., Hagadorn J.W. (2010). The Fossil Record of Cnidarian Medusae. Palaeoworld.

[9] David C.N., Özbek S., Adamczyk P., Meier S., Pauly B., Chapman J., Hwang J.S., Gojobori T., Holstein T.W. (2008). Evolution of Complex Structures: Minicollagens Shape the Cnidarian Nematocyst. Trends Genet..

[10] Nüchter T., Benoit M., Engel U., Özbek S., Holstein T.W. (2006). Nanosecond-Scale Kinetics of Nematocyst Discharge. Curr. Biol..

[11] Amreen Nisa S., Vinu D., Krupakar P., Govindaraju K., Sharma D., Vivek R. (2021). Jellyfish Venom Proteins and Their Pharmacological Potentials: A Review. Int. J. Biol. Macromol..

[12] Kallert D.M., Ponader S., Eszterbauer E., El-Matbouli M., Haas W. (2007). Myxozoan Transmission via Actinospores: New Insights into Mechanisms and Adaptations for Host Invasion. Parasitology.

[13] Roop J.I., Chang K.C., Brem R.B. (2016). Polygenic Evolution of a Sugar Specialization Trade-off in Yeast. Nature.

[14] Zhao H., Zhou Y., Pinto C.M., Charles-Dominique P., Galindo-González J., Zhang S., Zhang J. (2010). Evolution of the Sweet Taste Receptor Gene Tas1r2 in Bats. Mol. Biol. Evol..

[15] Wainwright P.C., Smith W.L., Price S.A., Tang K.L., Sparks J.S., Ferry L.A., Kuhn K.L., Eytan R.I., Near T.J. (2012). The Evolution of Pharyngognathy: A Phylogenetic and Functional Appraisal of the Pharyngeal Jaw Key Innovation in Labroid Fishes and Beyond. Syst. Biol..

[16] Drummond C.S., Eastwood R.J., Miotto S.T., Hughes C.E. (2012). Multiple Continental Radiations and Correlates of Diversification in *Lupinus* (Leguminosae): Testing for Key Innovation with Incomplete Taxon Sampling. Syst. Biol..

[17] Cracraft J. (1990). The Origin of Evolutionary Novelties: Pattern and Process at Different Hierarchical Levels. Evol. Innov..

[18] Balasubramanian P.G., Beckmann A., Warnken U., Schnolzer M., Schuler A., Bornberg-Bauer E., Holstein T.W., Ozbek S. (2012). Proteome of *Hydra* Nematocyst. J. Biol. Chem..

[19] Moran Y., Praher D., Schlesinger A., Ayalon A., Tal Y., Technau U. (2013). Analysis of Soluble Protein Contents from the Nematocysts of a Model Sea Anemone Sheds Light on Venom Evolution. Mar. Biotechnol..

[20] Gacesa R., Chung R., Dunn S.R., Weston A.J., Jaimes-Becerra A., Marques A.C., Morandini A.C., Hranueli D., Starcevic A., Ward M. (2015). Gene Duplications Are Extensive and Contribute Significantly to the Toxic Proteome of Nematocysts Isolated from *Acropora Digitifera* (Cnidaria: Anthozoa: Scleractinia). BMC Genom..

[21] Rachamim T., Morgenstern D., Aharonovich D., Brekhman V., Lotan T., Sher D. (2015). The Dynamically Evolving Nematocyst Content of an Anthozoan, a Scyphozoan, and a Hydrozoan. Mol. Biol. Evol..

[22] Jaimes-Becerra A., Gacesa R., Doonan L.B., Hartigan A., Marques A.C., Okamura B., Long P.F. (2019). “Beyond Primary Sequence”—Proteomic Data Reveal Complex Toxins in Cnidarian Venoms. Integr. Comp. Biol..

[23] Weston A.J., Chung R., Dunlap W.C., Morandini A.C., Marques A.C., Moura-da-Silva A.M., Ward M., Padilla G., da Silva L.F., Andreakis N. (2013). Proteomic Characterisation of Toxins Isolated from Nematocysts of the South Atlantic Jellyfish *Olindias Sambaquiensis*. Toxicon.

[24] Brinkman D.L., Jia X., Potriquet J., Kumar D., Dash D., Kvaskoff D., Mulvenna J. (2015). Transcriptome and Venom Proteome of the Box Jellyfish *Chironex Fleckeri*. BMC Genom..

[25] Jaimes-Becerra A., Chung R., Morandini A.C., Weston A.J., Padilla G., Gacesa R., Ward M., Long P.F., Marques A.C. (2017). Comparative Proteomics Reveals Recruitment Patterns of Some Protein Families in the Venoms of Cnidaria. Toxicon.

[26] Li R., Yu H., Yue Y., Liu S., Xing R., Chen X., Li P. (2016). Combined Proteomics and Transcriptomics Identifies Sting-Related Toxins of Jellyfish *Cyanea Nozakii*. J. Proteom..

[27] Ponce D., Brinkman D., Potriquet J., Mulvenna J. (2016). Tentacle Transcriptome and Venom Proteome of the Pacific Sea Nettle, *Chrysaora Fuscescens* (Cnidaria: Scyphozoa). Toxins.

[28] Yue Y., Yu H., Li R., Xing R., Liu S., Li K., Wang X., Chen X., Li P. (2017). Functional Elucidation of *Nemopilema Nomurai* and *Cyanea Nozakii* Nematocyst Venoms’ Lytic Activity Using Mass Spectrometry and Zymography. Toxins.

[29] Ponce-Garcia D.P. (2017). Transcriptomic, Proteomic and Biological Analyses of Venom Proteins from Two *Chrysaora* Jellyfish. Ph.D. Thesis.

[30] Wang C., Wang B., Wang B., Wang Q., Liu G., Wang T., He Q., Zhang L. (2019). Unique Diversity of Sting-Related Toxins Based on Transcriptomic and Proteomic Analysis of the Jellyfish *Cyanea Capillata* and *Nemopilema Nomurai* (Cnidaria: Scyphozoa). J. Proteome Res..

[31] Leung T.C.N., Qu Z., Nong W., Hui J.H.L., Ngai S.M. (2020). Proteomic Analysis of the Venom of Jellyfishes *Rhopilema Esculentum* and *Sanderia Malayensis*. Mar. Drugs.

[32] Piriatinskiy G., Atkinson S.D., Park S., Morgenstern D., Brekhman V., Yossifon G., Bartholomew J.L., Lotan T. (2017). Functional and Proteomic Analysis of *Ceratonova Shasta* (Cnidaria: Myxozoa) Polar Capsules Reveals Adaptations to Parasitism. Sci. Rep..

[33] Gavelis G.S., Wakeman K.C., Tillmann U., Ripken C., Mitarai S., Herranz M., Özbek S., Holstein T., Keeling P.J., Leander B.S. (2017). Microbial Arms Race: Ballistic “Nematocysts” in Dinoflagellates Represent a New Extreme in Organelle Complexity. Sci. Adv..

[34] Lom J., Dyková I. (2013). Myxozoan Genera: Definition and Notes on Taxonomy, Life-Cycle Terminology and Pathogenic Species. Folia Parasitol. (Praha).

[35] Chang E.S., Neuhof M., Rubinstein N.D., Diamant A., Philippe H., Huchon D., Cartwright P. (2015). Genomic Insights into the Evolutionary Origin of Myxozoa within Cnidaria. Proc. Natl. Acad. Sci. USA.

[36] Okamura B., Gruhl A., De Baets K., Huntley J.W. (2021). Evolution, Origins and Diversification of Parasitic Cnidarians. The Evolution and Fossil Record of Parasitism: Identification and Macroevolution of Parasites.

[37] Stilwell J.M., Griffin M.J., Rosser T.G., Mohammed H.H., Sidor I.F., Camus A.C. (2020). Insights into Myxozoan Composition and Physiology Revealed by Histochemical Properties of Myxospores. J. Fish Dis..

[38] Americus B., Austin B.M., Lotan T., Bartholomew J.L., Atkinson S.D. (2020). In Vitro and In Vivo Assays Reveal That Cations Affect Nematocyst Discharge in *Myxobolus cerebralis* (Cnidaria: Myxozoa). Parasitology.

[39] Cannon Q., Wagner E. (2003). Comparison of Discharge Mechanisms of Cnidarian Cnidae and Myxozoan Polar Capsules. Rev. Fish. Sci..

[40] Ben-David J., Atkinson S.D., Pollak Y., Yossifon G., Shavit U., Bartholomew J.L., Lotan T. (2016). Myxozoan Polar Tubules Display Structural and Functional Variation. Parasit. Vectors.

[41] Americus B., Lotan T., Bartholomew J.L., Atkinson S.D. (2020). A Comparison of the Structure and Function of Nematocysts in Free-Living and Parasitic Cnidarians (Myxozoa). Int. J. Parasitol..

[42] Naldoni J., Zatti S.A., da Silva M.R.M., Maia A.A.M., Adriano E.A. (2019). Morphological, Ultrastructural, and Phylogenetic Analysis of Two Novel *Myxobolus* Species (Cnidaria: Myxosporea) Parasitizing Bryconid Fish from São Francisco River, Brazil. Parasitol. Int..

[43] Lom J., Arthur J.R. (1989). A Guideline for the Preparation of Species Descriptions in Myxosporea. J. Fish Dis..

[44] Council N.R. (2010). Guide for the Care and Use of Laboratory Animals.

[45] Guo Q., Liu Y., Zhai Y., Gu Z. (2020). A Fast and Effective Method for Dissecting Parasitic Spores: Myxozoans as an Example. J. Exp. Biol..

[46] Bolger A.M., Lohse M., Usadel B. (2014). Trimmomatic: A Flexible Trimmer for Illumina Sequence Data. Bioinformatics.

[47] Grabherr M.G., Haas B.J., Yassour M., Levin J.Z., Thompson D.A., Amit I., Adiconis X., Fan L., Raychowdhury R., Zeng Q. (2011). Full-Length Transcriptome Assembly from RNA-Seq Data without a Reference Genome. Nat. Biotechnol..

[48] Li W., Godzik A. (2006). Cd-Hit: A Fast Program for Clustering and Comparing Large Sets of Protein or Nucleotide Sequences. Bioinformatics.

[49] Pertea G., Huang X., Liang F., Antonescu V., Sultana R., Karamycheva S., Lee Y., White J., Cheung F., Parvizi B. (2003). TIGR Gene Indices Clustering Tools (TGICL): A Software System for Fast Clustering of Large EST Datasets. Bioinformatics.

[50] Luo R., Liu B., Xie Y., Li Z., Huang W., Yuan J., He G., Chen Y., Pan Q., Liu Y. (2012). SOAPdenovo2: An Empirically Improved Memory-Efficient Short-Read de Novo Assembler. Gigascience.

[51] Guo Q., Li D., Zhai Y., Gu Z. (2020). CCPRD: A Novel Analytical Framework for the Comprehensive Proteomic Reference Database Construction of Nonmodel Organisms. ACS Omega.

[52] Camacho C., Coulouris G., Avagyan V., Ma N., Papadopoulos J., Bealer K., Madden T.L. (2009). BLAST+: Architecture and Applications. BMC Bioinform..

[53] Laetsch D.R., Blaxter M.L. (2017). BlobTools: Interrogation of Genome Assemblies. F1000Research.

[54] Lomsadze A., Burns P.D., Borodovsky M. (2014). Integration of Mapped RNA-Seq Reads into Automatic Training of Eukaryotic Gene Finding Algorithm. Nucl. Acids Res..

[55] Stanke M., Morgenstern B. (2005). AUGUSTUS: A Web Server for Gene Prediction in Eukaryotes That Allows User-Defined Constraints. Nucl. Acids Res..

[56] Korf I. (2004). Gene Finding in Novel Genomes. BMC Bioinform..

[57] Birney E., Clamp M., Durbin R. (2004). GeneWise and Genomewise. Genome Res..

[58] Haas B.J., Delcher A.L., Mount S.M., Wortman J.R., Smith R.K., Hannick L.I., Maiti R., Ronning C.M., Rusch D.B., Town C.D. (2003). Improving the *Arabidopsis* Genome Annotation Using Maximal Transcript Alignment Assemblies. Nucl. Acids Res..

[59] Haas B.J., Papanicolaou A., Yassour M., Grabherr M., Blood P.D., Bowden J., Couger M.B., Eccles D., Li B., Lieber M. (2013). De Novo Transcript Sequence Reconstruction from RNA-Seq Using the Trinity Platform for Reference Generation and Analysis. Nat. Protoc..

[60] Tang S., Lomsadze A., Borodovsky M. (2015). Identification of Protein Coding Regions in RNA Transcripts. Nucl. Acids Res..

[61] Rice P., Longden I., Bleasby A. (2000). EMBOSS: The European Molecular Biology Open Software Suite. Trends Genet..

[62] Tyanova S., Temu T., Cox J. (2016). The MaxQuant Computational Platform for Mass Spectrometry-Based Shotgun Proteomics. Nat. Protoc..

[63] Schwanhäusser B., Busse D., Li N., Dittmar G., Schuchhardt J., Wolf J., Chen W., Selbach M. (2011). Global Quantification of Mammalian Gene Expression Control. Nature.

[64] Conesa A., Götz S., García-Gómez J.M., Terol J., Talón M., Robles M. (2005). Blast2GO: A Universal Tool for Annotation, Visualization and Analysis in Functional Genomics Research. Bioinformatics.

[65] Finn R.D., Attwood T.K., Babbitt P.C., Bateman A., Bork P., Bridge A.J., Chang H.-Y., Dosztányi Z., El-Gebali S., Fraser M. (2016). InterPro in 2017—Beyond Protein Family and Domain Annotations. Nucl. Acids Res..

[66] Li L., Stoeckert C.J., Roos D.S. (2003). OrthoMCL: Identification of Ortholog Groups for Eukaryotic Genomes. Genome Res..

[67] Chen H., Boutros P.C. (2011). VennDiagram: A Package for the Generation of Highly-Customizable Venn and Euler Diagrams in R. BMC Bioinform..

[68] Conway J.R., Lex A., Gehlenborg N. (2017). UpSetR: An R Package for the Visualization of Intersecting Sets and Their Properties. Bioinformatics.

[69] Darzentas N. (2010). Circoletto: Visualizing Sequence Similarity with Circos. Bioinformatics.

[70] Krzywinski M., Schein J., Birol I., Connors J., Gascoyne R., Horsman D., Jones S.J., Marra M.A. (2009). Circos: An Information Aesthetic for Comparative Genomics. Genome Res..

[71] Gacesa R., Barlow D.J., Long P.F. (2016). Machine Learning Can Differentiate Venom Toxins from Other Proteins Having Non-Toxic Physiological Functions. Peer J. Comput. Sci..

[72] Jungo F., Bougueleret L., Xenarios I., Poux S. (2012). The UniProtKB/Swiss-Prot Tox-Prot Program: A Central Hub of Integrated Venom Protein Data. Toxicon.

[73] Ebersberger I., Strauss S., von Haeseler A. (2009). HaMStR: Profile Hidden Markov Model Based Search for Orthologs in ESTs. BMC Evol. Biol..

[74] Cannon J.T., Vellutini B.C., Smith J., Ronquist F., Jondelius U., Hejnol A. (2016). Xenacoelomorpha Is the Sister Group to Nephrozoa. Nature.

[75] Capella-Gutiérrez S., Silla-Martínez J.M., Gabaldón T. (2009). TrimAl: A Tool for Automated Alignment Trimming in Large-Scale Phylogenetic Analyses. Bioinformatics.

[76] Kocot K.M., Citarella M.R., Moroz L.L., Halanych K.M. (2013). PhyloTreePruner: A Phylogenetic Tree-Based Approach for Selection of Orthologous Sequences for Phylogenomics. Evol. Bioinform..

[77] Price M.N., Dehal P.S., Arkin A.P. (2010). FastTree 2–Approximately Maximum-Likelihood Trees for Large Alignments. PLoS ONE.

[78] Struck T.H. (2014). TreSpEx-Detection of Misleading Signal in Phylogenetic Reconstructions Based on Tree Information. Evol. Bioinform..

[79] Finn R.D., Clements J., Eddy S.R. (2011). HMMER Web Server: Interactive Sequence Similarity Searching. Nucl. Acids Res..

[80] Katoh K., Standley D.M. (2013). MAFFT Multiple Sequence Alignment Software Version 7: Improvements in Performance and Usability. Mol. Biol. Evol..

[81] Castresana J. (2000). Selection of Conserved Blocks from Multiple Alignments for Their Use in Phylogenetic Analysis. Mol. Biol. Evol..

[82] Nguyen L.-T., Schmidt H.A., von Haeseler A., Minh B.Q. (2014). IQ-TREE: A Fast and Effective Stochastic Algorithm for Estimating Maximum-Likelihood Phylogenies. Mol. Biol. Evol..

[83] Roure B., Rodriguez-Ezpeleta N., Philippe H. (2007). SCaFoS: A Tool for Selection, Concatenation and Fusion of Sequences for Phylogenomics. BMC Evol. Biol..

[84] Stamatakis A. (2014). RAxML Version 8: A Tool for Phylogenetic Analysis and Post-Analysis of Large Phylogenies. Bioinformatics.

[85] Lartillot N., Lepage T., Blanquart S. (2009). PhyloBayes 3: A Bayesian Software Package for Phylogenetic Reconstruction and Molecular Dating. Bioinformatics.

[86] Shimodaira H. (2002). An Approximately Unbiased Test of Phylogenetic Tree Selection. Syst. Biol..

[87] Brown M.W., Sharpe S.C., Silberman J.D., Heiss A.A., Lang B.F., Simpson A.G., Roger A.J. (2013). Phylogenomics Demonstrates That Breviate Flagellates Are Related to Opisthokonts and Apusomonads. Proc. R. Soc. Lond. B Biol. Sci..

[88] Susko E., Field C., Blouin C., Roger A.J. (2003). Estimation of Rates-across-Sites Distributions in Phylogenetic Substitution Models. Syst. Biol..

[89] Sanderson M.J. (2003). R8s: Inferring Absolute Rates of Molecular Evolution and Divergence Times in the Absence of a Molecular Clock. Bioinformatics.

[90] Park E., Hwang D.-S., Lee J.-S., Song J.-I., Seo T.-K., Won Y.-J. (2012). Estimation of Divergence Times in Cnidarian Evolution Based on Mitochondrial Protein-Coding Genes and the Fossil Record. Mol. Phylogenet. Evol..

[91] Schwentner M., Bosch T.C.G. (2015). Revisiting the Age, Evolutionary History and Species Level Diversity of the Genus Hydra (Cnidaria: Hydrozoa). Mol. Phylogenet. Evol..

[92] Kosakovsky Pond S.L., Poon A.F.Y., Velazquez R., Weaver S., Hepler N.L., Murrell B., Shank S.D., Magalis B.R., Bouvier D., Nekrutenko A. (2020). HyPhy 2.5—A Customizable Platform for Evolutionary Hypothesis Testing Using Phylogenies. Mol. Biol. Evol..

[93] Shinzato C., Shoguchi E., Kawashima T., Hamada M., Hisata K., Tanaka M., Fujie M., Fujiwara M., Koyanagi R., Ikuta T. (2011). Using the *Acropora Digitifera* Genome to Understand Coral Responses to Environmental Change. Nature.

[94] Chapman J.A., Kirkness E.F., Simakov O., Hampson S.E., Mitros T., Weinmaier T., Rattei T., Balasubramanian P.G., Borman J., Busam D. (2010). The Dynamic Genome of *Hydra*. Nature.

[95] Prada C., Hanna B., Budd A.F., Woodley C.M., Schmutz J., Grimwood J., Iglesias-Prieto R., Pandolfi J.M., Levitan D., Johnson K.G. (2016). Empty Niches after Extinctions Increase Population Sizes of Modern Corals. Curr. Biol..

[96] Voolstra C.R., Li Y., Liew Y.J., Baumgarten S., Zoccola D., Flot J.-F., Tambutté S., Allemand D., Aranda M. (2017). Comparative Analysis of the Genomes of *Stylophora Pistillata* and *Acropora Digitifera* Provides Evidence for Extensive Differences between Species of Corals. Sci. Rep..

[97] Yang Y., Xiong J., Zhou Z., Huo F., Miao W., Ran C., Liu Y., Zhang J., Feng J., Wang M. (2014). The Genome of the Myxosporean *Thelohanellus Kitauei* Shows Adaptations to Nutrient Acquisition within Its Fish Host. Genome Biol. Evol..

[98] Baumgarten S., Simakov O., Esherick L.Y., Liew Y.J., Lehnert E.M., Michell C.T., Li Y., Hambleton E.A., Guse A., Oates M.E. (2015). The Genome of *Aiptasia*, a Sea Anemone Model for Coral Symbiosis. Proc. Natl. Acad. Sci. USA.

[99] Putnam N.H., Srivastava M., Hellsten U., Dirks B., Chapman J., Salamov A., Terry A., Shapiro H., Lindquist E., Kapitonov V.V. (2007). Sea Anemone Genome Reveals Ancestral Eumetazoan Gene Repertoire and Genomic Organization. Science.

[100] Enard D., Cai L., Gwennap C., Petrov D.A. (2016). Viruses Are a Dominant Driver of Protein Adaptation in Mammals. Elife.

[101] Yang Z. (2007). PAML 4: Phylogenetic Analysis by Maximum Likelihood. Mol. Biol. Evol..

[102] Sasikumar A.N., Perez W.B., Kinzy T.G. (2012). The Many Roles of the Eukaryotic Elongation Factor 1 Complex. Wiley Interdiscip. Rev. RNA.

[103] Talbert P.B., Henikoff S. (2010). Histone Variants—Ancient Wrap Artists of the Epigenome. Nat. Rev. Mol. Cell Biol..

[104] Whitley D., Goldberg S.P., Jordan W.D. (1999). Heat Shock Proteins: A Review of the Molecular Chaperones. J. Vasc. Surg..

[105] Hwang J.S., Takaku Y., Momose T., Adamczyk P., Özbek S., Ikeo K., Khalturin K., Hemmrich G., Bosch T.C., Holstein T.W. (2010). Nematogalectin, a Nematocyst Protein with GlyXY and Galectin Domains, Demonstrates Nematocyte-Specific Alternative Splicing in *Hydra*. Proc. Natl. Acad. Sci. USA.

[106] Draizen E.J., Shaytan A.K., Mariño-Ramírez L., Talbert P.B., Landsman D., Panchenko A.R. (2016). HistoneDB 2.0: A Histone Database with Variants—An Integrated Resource to Explore Histones and Their Variants. Database.

[107] Kayal E., Bentlage B., Sabrina Pankey M., Ohdera A.H., Medina M., Plachetzki D.C., Collins A.G., Ryan J.F. (2018). Phylogenomics Provides a Robust Topology of the Major Cnidarian Lineages and Insights on the Origins of Key Organismal Traits. BMC Evol. Biol..

[108] Ashwood L.M., Norton R.S., Undheim E.A.B., Hurwood D.A., Prentis P.J. (2020). Characterising Functional Venom Profiles of Anthozoans and Medusozoans within Their Ecological Context. Mar. Drugs.

[109] Jouiaei M., Yanagihara A.A., Madio B., Nevalainen T.J., Alewood P.F., Fry B.G. (2015). Ancient Venom Systems: A Review on Cnidaria Toxins. Toxins.

[110] D’Ambra I., Lauritano C. (2020). A Review of Toxins from Cnidaria. Mar. Drugs.

[111] Sunagar K., Moran Y. (2015). The Rise and Fall of an Evolutionary Innovation: Contrasting Strategies of Venom Evolution in Ancient and Young Animals. PLoS Genet..

[112] Klompen A.M.L., Kayal E., Collins A.G., Cartwright P. (2021). Phylogenetic and Selection Analysis of an Expanded Family of Putatively Pore-Forming Jellyfish Toxins (Cnidaria: Medusozoa). Genome Biol. Evol..

[113] Shpirer E., Chang E.S., Diamant A., Rubinstein N., Cartwright P., Huchon D. (2014). Diversity and Evolution of Myxozoan Minicollagens and Nematogalectins. BMC Evol. Biol..

[114] Kyslík J., Kosakyan A., Nenarokov S., Holzer A.S., Fiala I. (2021). The Myxozoan Minicollagen Gene Repertoire Was Not Simplified by the Parasitic Lifestyle: Computational Identification of a Novel Myxozoan Minicollagen Gene. BMC Genom..

[115] Ganot P., Moya A., Magnone V., Allemand D., Furla P., Sabourault C. (2011). Adaptations to Endosymbiosis in a Cnidarian-Dinoflagellate Association: Differential Gene Expression and Specific Gene Duplications. PLoS Genet..

[116] Liu R., Ochman H. (2007). Stepwise Formation of the Bacterial Flagellar System. Proc. Natl. Acad. Sci. USA.

[117] Arivalagan J., Yarra T., Marie B., Sleight V.A., Duvernois-Berthet E., Clark M.S., Marie A., Berland S. (2017). Insights from the Shell Proteome: Biomineralization to Adaptation. Mol. Biol. Evol..

[118] Abby S.S., Rocha E.P.C. (2012). The Non-Flagellar Type III Secretion System Evolved from the Bacterial Flagellum and Diversified into Host-Cell Adapted Systems. PLoS Genet..

[119] Nogales M., Padrón B., Padilla D.P., Nieves C., Marrero P. (2009). Germination Patterns throughout an Insular Altitudinal Gradient: The Case of the Macaronesian Endemic Plant *Rubia Fruticosa* Ait. (Rubiaceae) in El Hierro (Canary Islands). Flora-Morphol. Distrib. Funct. Ecol. Plants.

[120] Cartwright P., Collins A. (2007). Fossils and Phylogenies: Integrating Multiple Lines of Evidence to Investigate the Origin of Early Major Metazoan Lineages. Integr. Comp. Biol..

[121] Peterson K.J., Lyons J.B., Nowak K.S., Takacs C.M., Wargo M.J., McPeek M.A. (2004). Estimating Metazoan Divergence Times with a Molecular Clock. Proc. Natl. Acad. Sci. USA.

[122] Waggoner B., Collins A.G. (2004). Reductio Ad Absurdum: Testing the Evolutionary Relationships of Ediacaran and Paleozoic Problematic Fossils Using Molecular Divergence Dates. J. Paleontol..

[123] Smith M.D., Wertheim J.O., Weaver S., Murrell B., Scheffler K., Kosakovsky Pond S.L. (2015). Less Is More: An Adaptive Branch-Site Random Effects Model for Efficient Detection of Episodic Diversifying Selection. Mol. Biol. Evol..

[124] Murrell B., Weaver S., Smith M.D., Wertheim J.O., Murrell S., Aylward A., Eren K., Pollner T., Martin D.P., Smith D.M. (2015). Gene-Wide Identification of Episodic Selection. Mol. Biol. Evol..

[125] Turing A.M. (1990). The Chemical Basis of Morphogenesis. Bull. Math. Biol..

[126] Von Neumann N., Burks A.W. (1966). Theory of Self-Reproducing Automata.

[127] Gierer A., Berking S., Bode H., David C.N., Flick K., Hansmann G., Schaller H., Trenkner E. (1972). Regeneration of *Hydra* from Reaggregated Cells. Nat. New Biol..

[128] Meinhardt H. (2012). Turing’s Theory of Morphogenesis of 1952 and the Subsequent Discovery of the Crucial Role of Local Self-Enhancement and Long-Range Inhibition. Interface Focus.

[129] Zhang R., Jin L., Zhang N., Petridis A.K., Eckert T., Scheiner-Bobis G., Bergmann M., Scheidig A., Schauer R., Yan M. (2019). The Sialic Acid-Dependent Nematocyst Discharge Process in Relation to Its Physical-Chemical Properties Is a Role Model for Nanomedical Diagnostic and Therapeutic Tools. Mar. Drugs.

[130] Perez-Riverol Y., Csordas A., Bai J., Bernal-Llinares M., Hewapathirana S., Kundu D.J., Inuganti A., Griss J., Mayer G., Eisenacher M. (2019). The PRIDE Database and Related Tools and Resources in 2019: Improving Support for Quantification Data. Nucl. Acids Res..

