# Supplementary material for: Quantitative Insights into the Contribution of Nematocysts to the Adaptive Success of Cnidarians Based on Proteomic Analysis
Source: Biology (Basel). 2022 Jan 7;11(1):91. doi: 10.3390/biology11010091 (PMC8773148; doi:10.3390/biology11010091)
Supplement: Supplementary file 1 [file biology-11-00091-s001.zip › Supplementary_materials-1502701-revised-v3.pdf]

# Quantitative insights into the contribution of nematocysts to the adaptive success of cnidarians based on proteomic analysis

Qingxiang Guo[1,2,3], Christopher M. Whipps[4], Yanhua Zhai[1,2,3], Dan Li[1,2,3], Zemao Gu[1,2,3*]

[1]Department of Aquatic Animal Medicine, College of Fisheries, Huazhong Agricultural University, Wuhan 430070, China; qingxiang.guo@outlook.com (Q.G.); zhaiyh@mail.hzau.edu.cn (Y.Z.); liidan@webmail.hzau.edu.cn (D.L.)

[2]Hubei Engineering Technology Research Center for Aquatic Animal Diseases Control and Prevention, Wuhan 430070, China

[3]Engineering Research Center of Green Development for Conventional Aquatic Biological Industry in the Yangtze River Economic Belt, Ministry of Education, Wuhan 430070, China

[4]SUNY-ESF, College of Environmental Science and Forestry, State University of New York, 246 Illick Hall, 1 Forestry Drive, Syracuse, NY 13210, USA; cwhipps@esf.edu

*Correspondence: guzemao@mail.hzau.edu.cn; Tel.: +86-027-8728-2114

## SUPPLEMENTARY MATERIALS

## Table of Contents:


| | |
|---|---|
| **Supplementary Figure** | Page 19 |
| **Supplementary Table** | Page 20 |

| Sample | T | M | H |
|---|---|---|---|
| Concentration | 0.2ug/ul | 0.2ug/ul | 0.3ug/ul |
| Volume | 800ul | 800ul | 800ul |

**Figure S1.** Protein quantitation of the isolated myxozoan nematocysts. T, *Thelohanellus kitauei*; M, *Myxobolus wulii*; H, *Myxobolus honghuensis*.

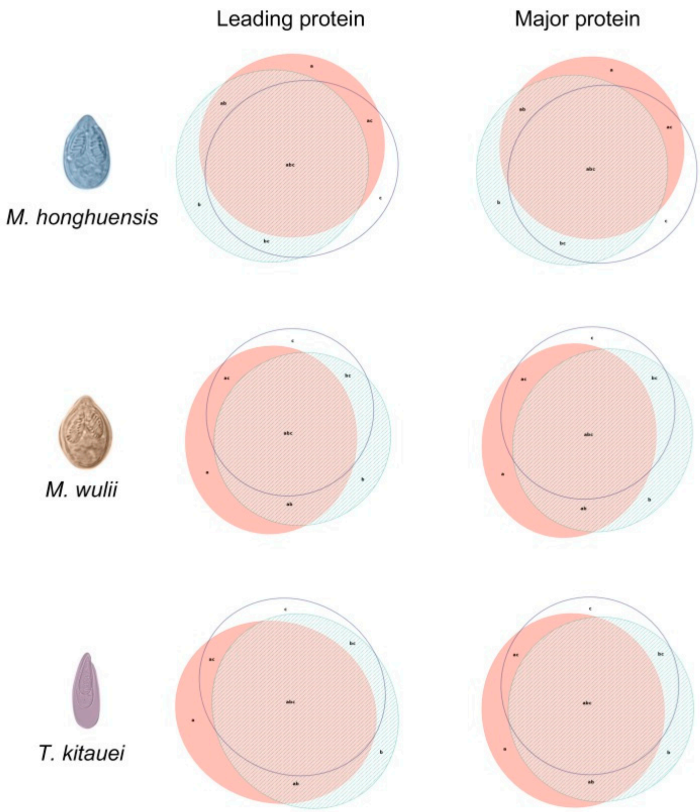


**Figure S2.** Comparisons between numbers of myxozoan nematocyst proteins identified in different selection criteria (leading protein based and major protein based) in three replicates of *Myxobolus honghuensis*, *Thelohanellus kitauei*, *Myxobolus wulii*.

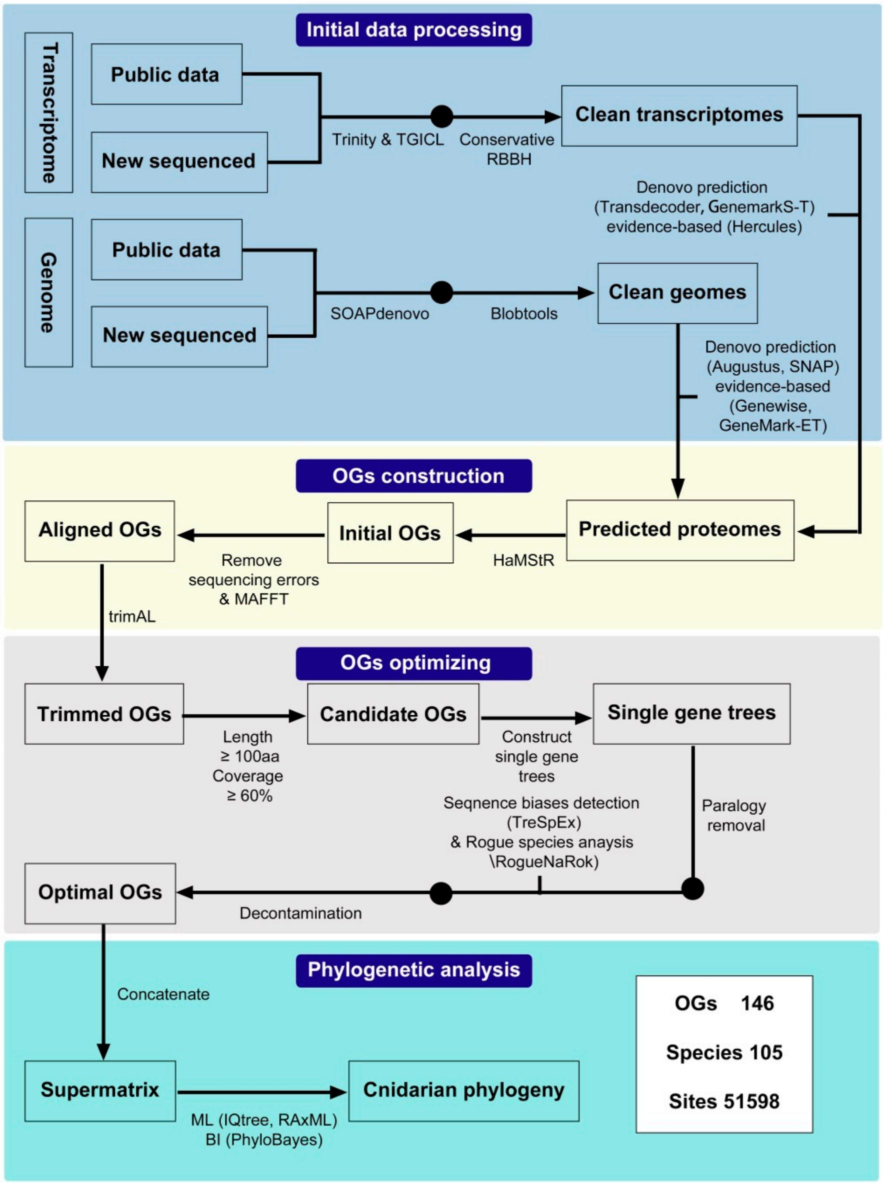


**Figure S3.** Flowchart of phylogenomic data analysis.

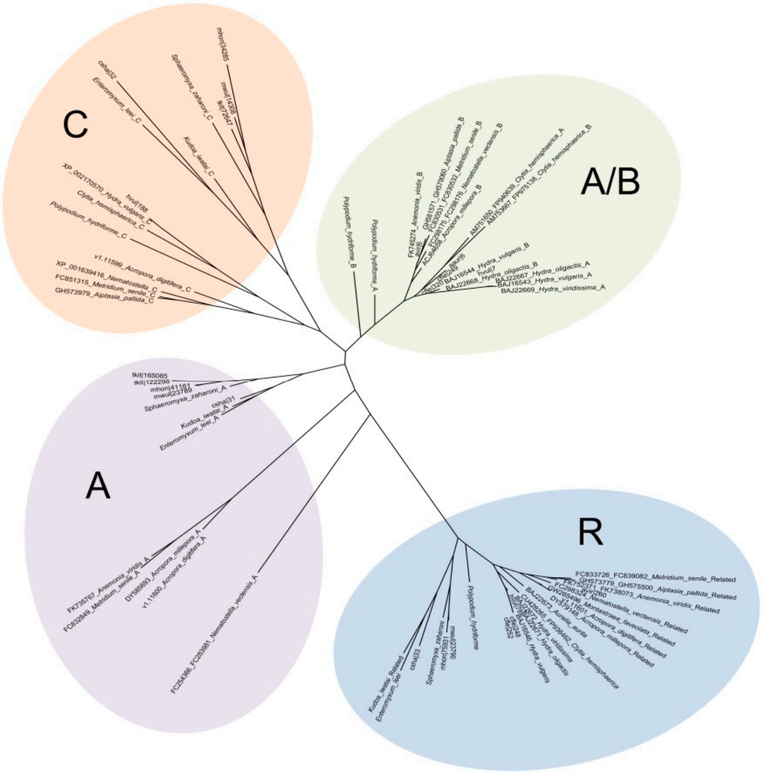


**Figure S4.** ML phylogenetic tree reconstructed based on the nematogalectin dataset of cnidarians. A, B ,C, R represent nematogalectin A, nematogalectin B, nematogalectin C and nematogalectin-related respectively.

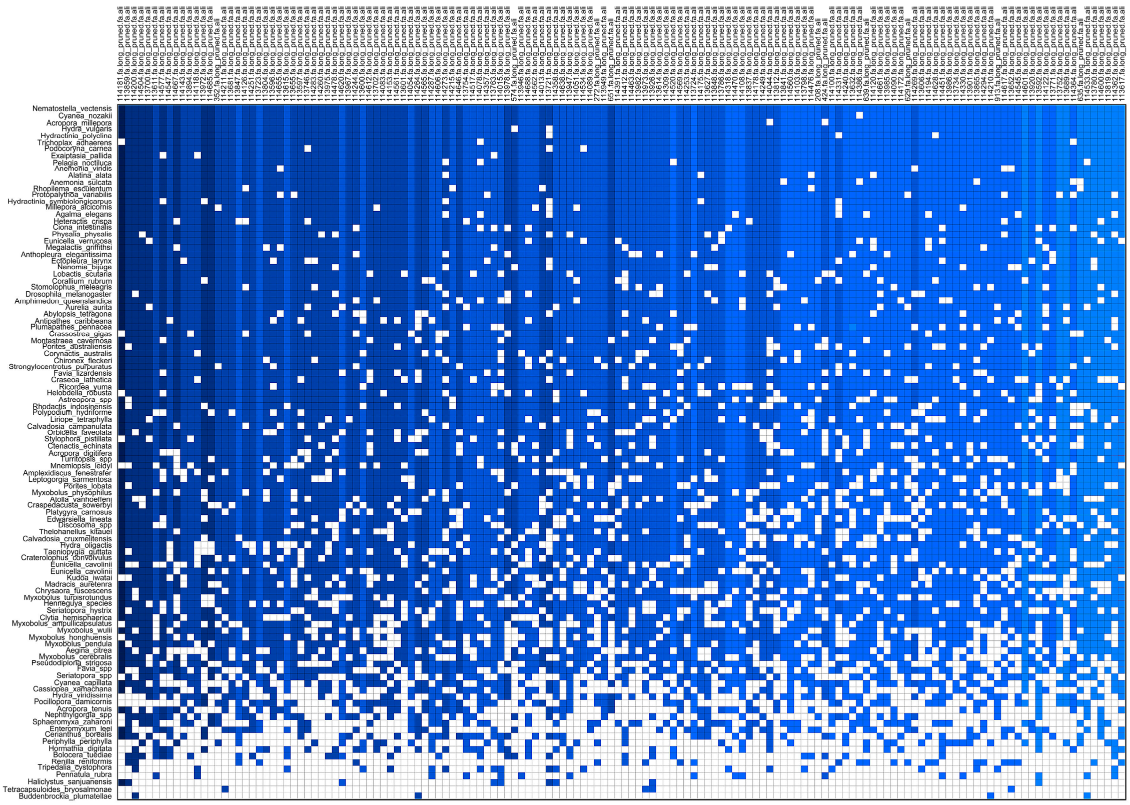

**Figure S5.** Visualization of the supermatrix for final phylogenomic constructions. Data matrix 4_103tx_146og for constructing main cnidarian phylogeny in Figure 5A.

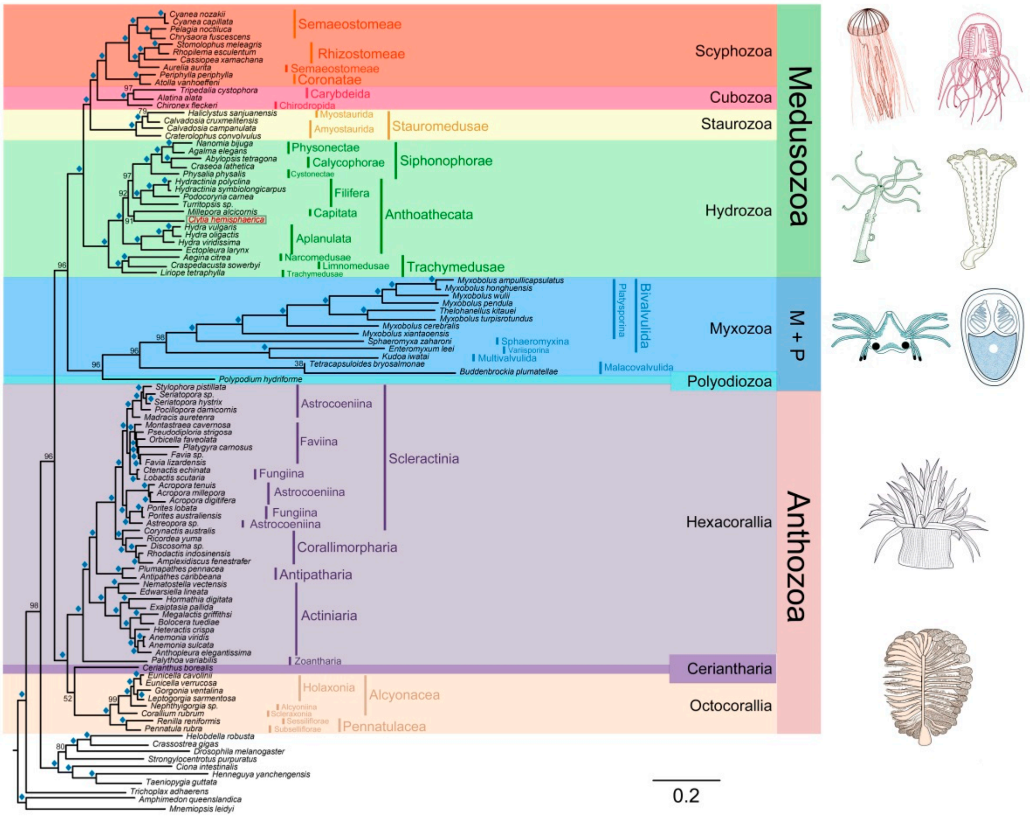


**Figure S6.** A cnidarian phylogram of the best ML tree inferred by IQ-TREE based on the initial amino acid dataset 0_untreat_105tx_146og (105 taxa and 51,598 amino-acid sites) with single-gene partitions under their best-fit models. Numbers beside nodes are supports values after 10,000 ultrafast bootstrap replicates. Diamond marks on branches correspond to 100% UF. Different clades are colored according to the classification.

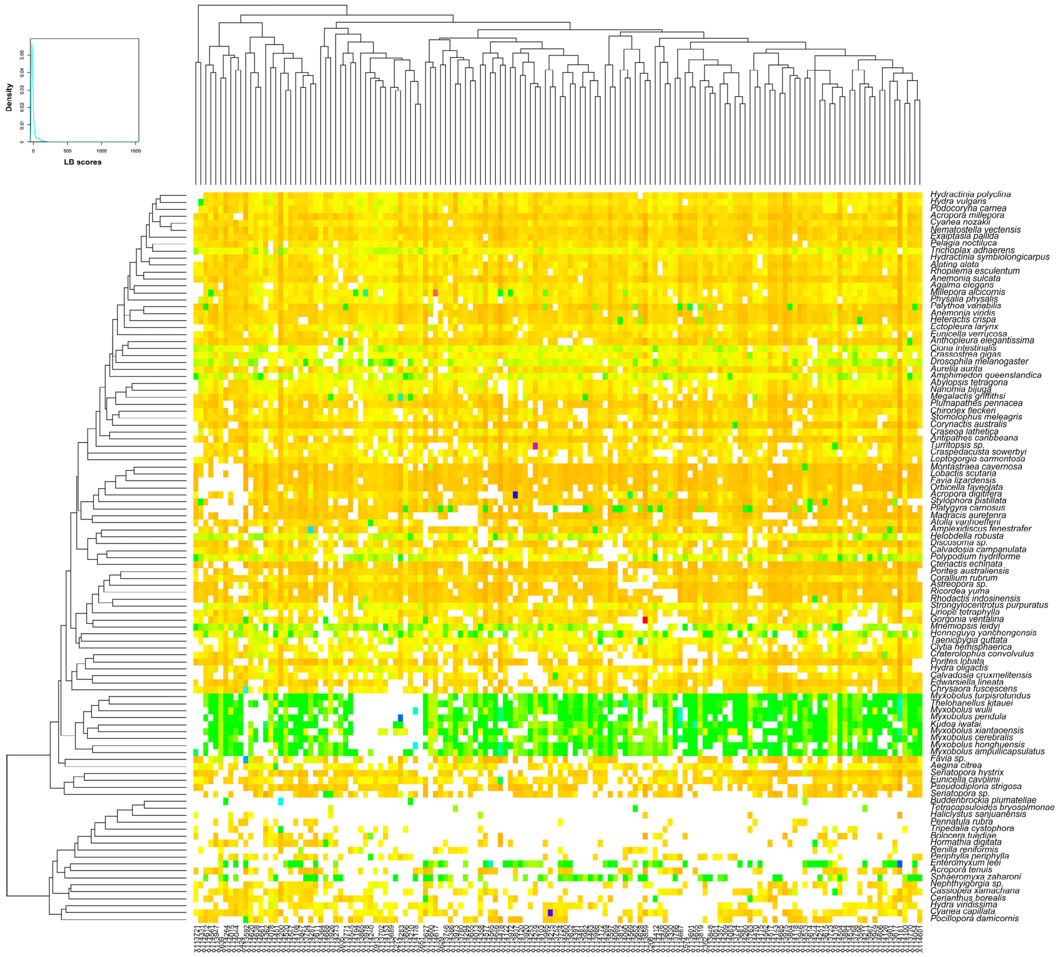


**Figure S7.** Heat map in combination with hierarchical clustering generated with R of the taxon-specific long-branch scores for the cnidarian phylogeny initial dataset 0_untreat_105tx_146og (105 taxa and 51,598 amino-acid sites). Row show taxa and columns genes. Color key and density plot of long-branch (LB) scores are shown in the upper left corner of the heat map. White cells in the map indicate that the taxon was lacking in that gene. Orange cells indicate that the taxon with low LB scores indicate that the taxon in that gene might be not affected by long-branch attraction. Cells with darker color (dark green, blue, purple and red) are with higher LB scores and indicate that the taxon in that gene might be a long-branch.

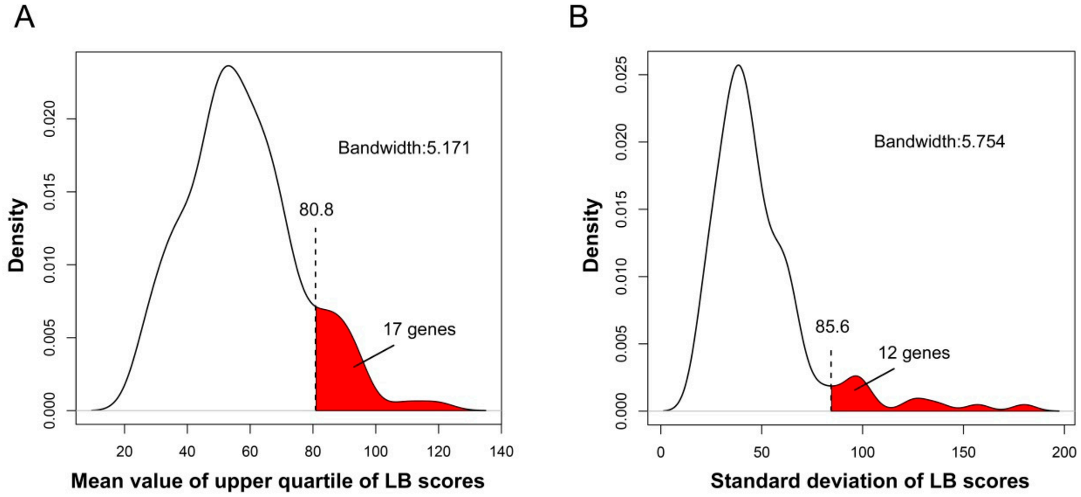


**Figure S8.** Density plots generated with R of different gene specific long-branch indices for the 146 genes of the cnidarian phylogeny initial dataset 0_untreat_105tx_146og. (A) Average of the upper quartile of LB scores representing the genes with the longest branches. (B) Standard deviation of LB scores measuring heterogeneity. Dashed line indicates starting shoulder value. Red areas in the right-hand side indicate deviations from the normal distribution and comprise genes might be long branches.

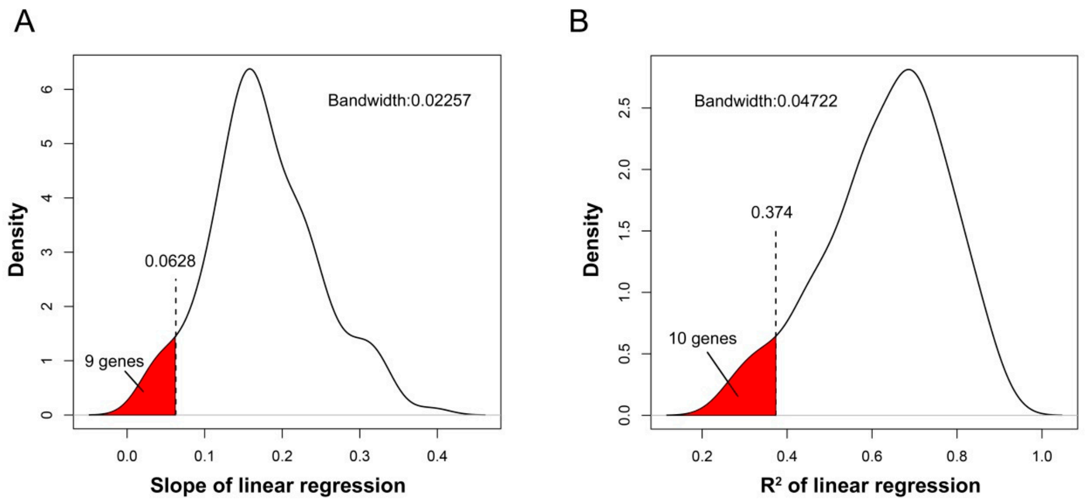


**Figure S9.** Density plots (distribution) of different gene-specific saturation indices for the 146 genes of the cnidarian phylogeny initial dataset 0_untreat_105tx_146og. (A) Slopes of the linear regression between patristic and uncorrected pairwise distances. (B) $R^2$ of the linear regression between patristic and uncorrected pairwise distances. Dashed line indicates starting shoulder value. Red areas in the left-hand side comprise genes that might be saturated.

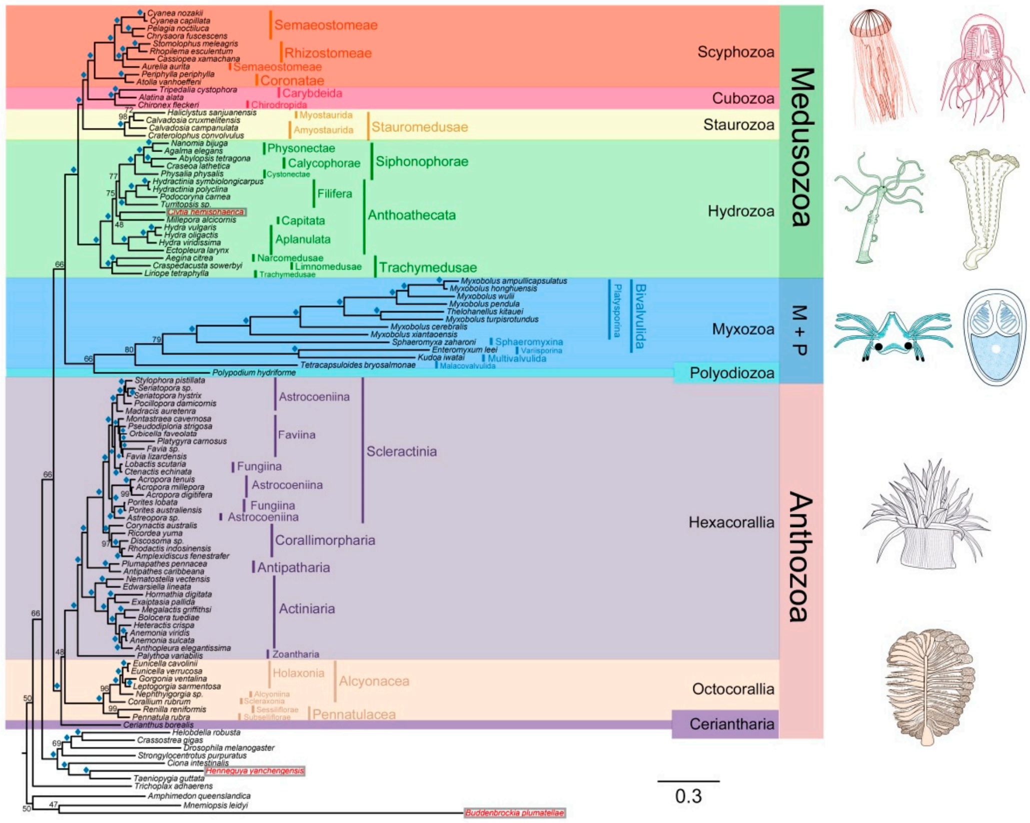

**Figure S10.** The cnidarian best ML tree inferred by RAxML based on the initial amino acid dataset 0_untreat_105tx_146og (105 taxa and 51,598 amino-acid sites) with single-gene partitions under the PROTGAMMAAUTO model. Numbers associated with branches are bootstrap values after 100 replicates. Diamond marks on branches correspond to 100% bp. Different clades are colored according to the classification.

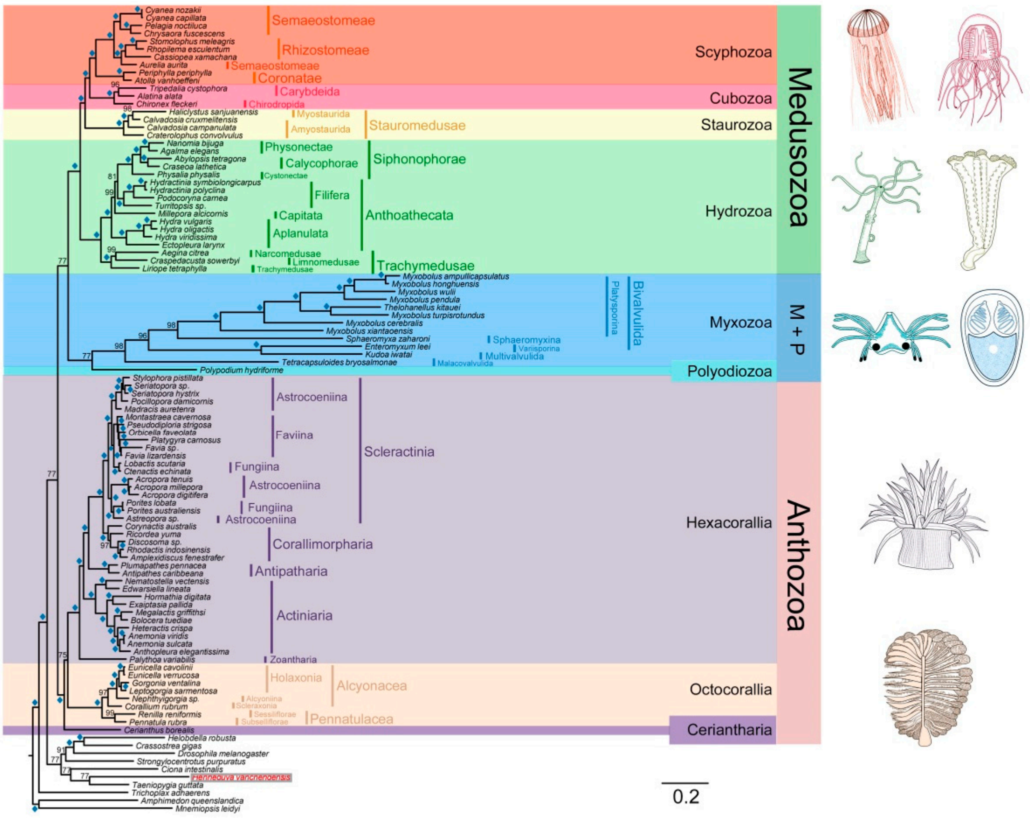


**Figure S11.** A cnidarian phylogram of the best ML tree inferred by IQ-TREE based on the amino acid dataset 1_optimized_103tx_121og (103 taxa and 42,573 amino-acid sites) with single-gene partitions under their best-fit models. Comparing to initial dataset, taxa *Buddenbrockia plumatellae* and *Clytia hemisphaerica* were excluded in this analysis. Genes with high long-branch scores and possibly saturated were identified by TreSpEx and also excluded. Numbers beside nodes are supports values after 10,000 ultrafast bootstrap replicates. Diamond marks on branches correspond to 100% UF. Different clades are colored according to the classification.

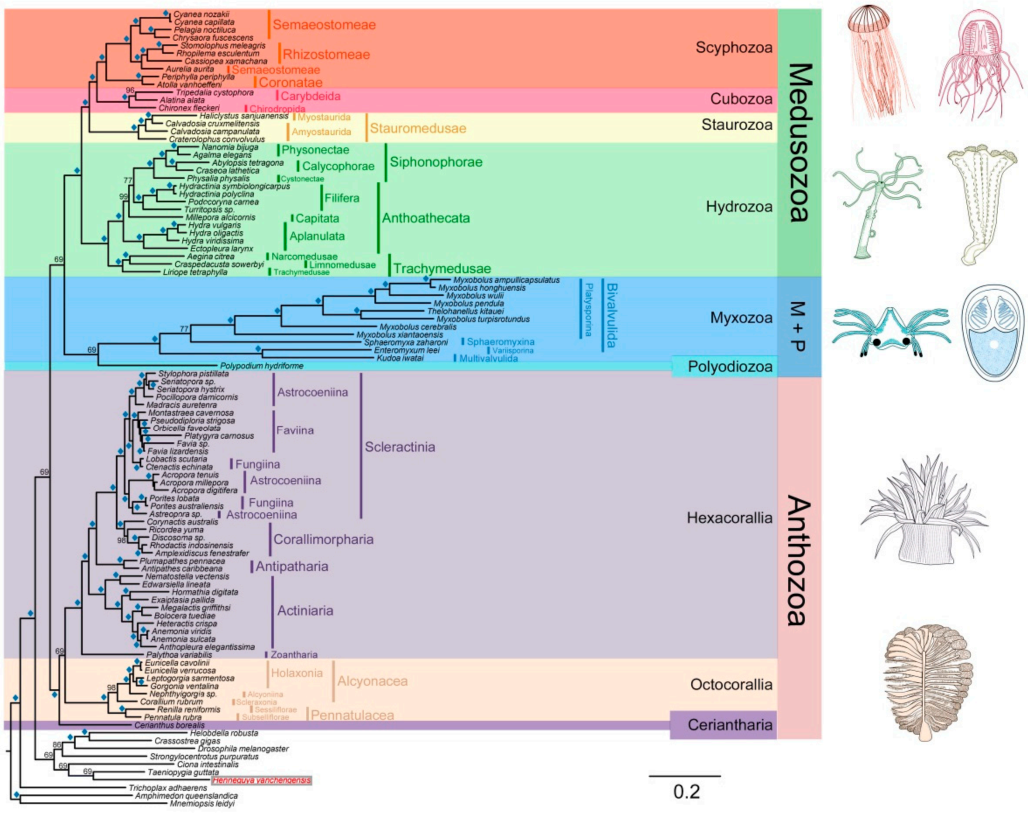


**Figure S12.** A cnidarian phylogram of the best ML tree inferred by IQ-TREE based on the amino acid dataset 2_optimized_102tx_121og (102 taxa and 42,573 amino-acid sites) with single-gene partitions under their best-fit models. Comparing to initial dataset, taxa *Tetracapsuloides bryosalmonae, Buddenbrockia plumatellae* and *Clytia hemisphaerica* were excluded in this analysis. Genes with high long-branch scores and possibly saturated were identified by TreSpEx and also excluded. Numbers beside nodes are supports values after 10,000 ultrafast bootstrap replicates. Diamond marks on branches correspond to 100% UF. Different clades are colored according to the classification.

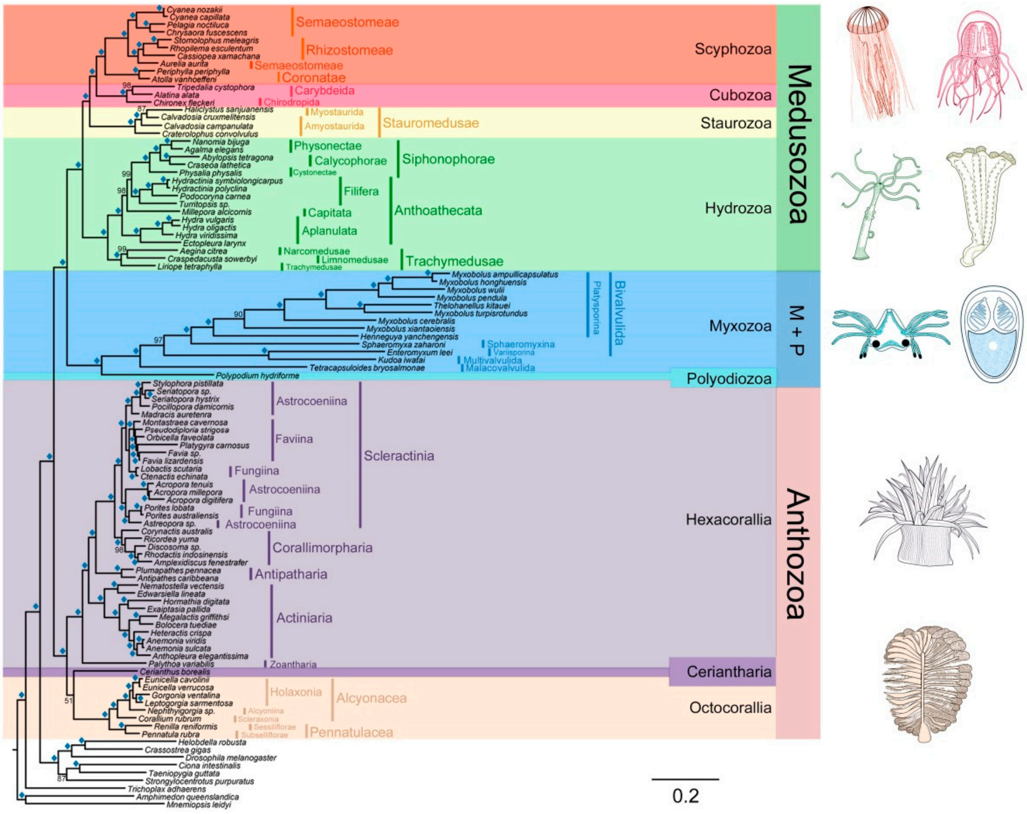


**Figure S13.** A cnidarian phylogram of the best ML tree inferred by IQ-TREE based on the main dataset 4_103tx_146og (103 taxa and 51,598 amino-acid sites) with single-gene partitions under their best-fit models. Numbers beside nodes are supports values after 10,000 ultrafast bootstrap replicates. Diamond marks on branches correspond to 100% UF. Different clades are colored according to the classification.

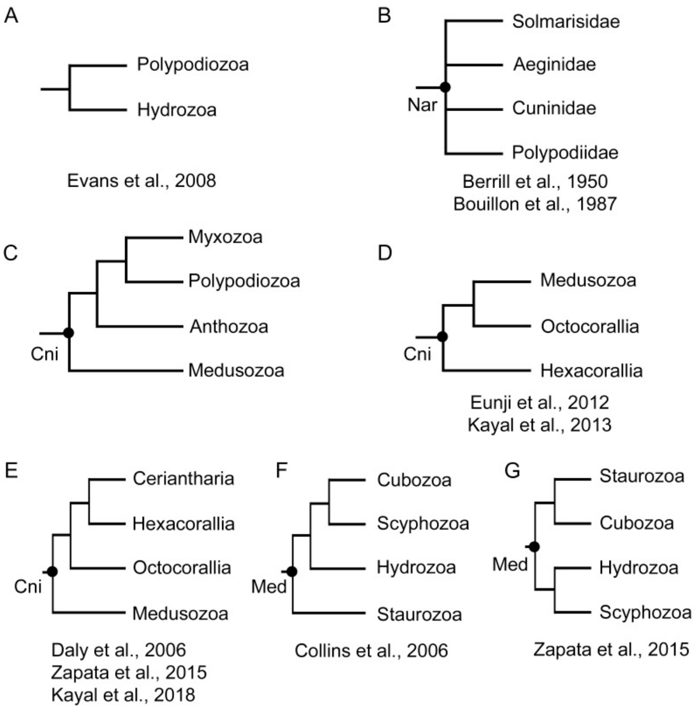


**Figure S14.** Alternative topologies among major lineages of cnidarians for AU test. Cni, Cnidaria; Med, Medusozoa; Nar, Narcomedusae.

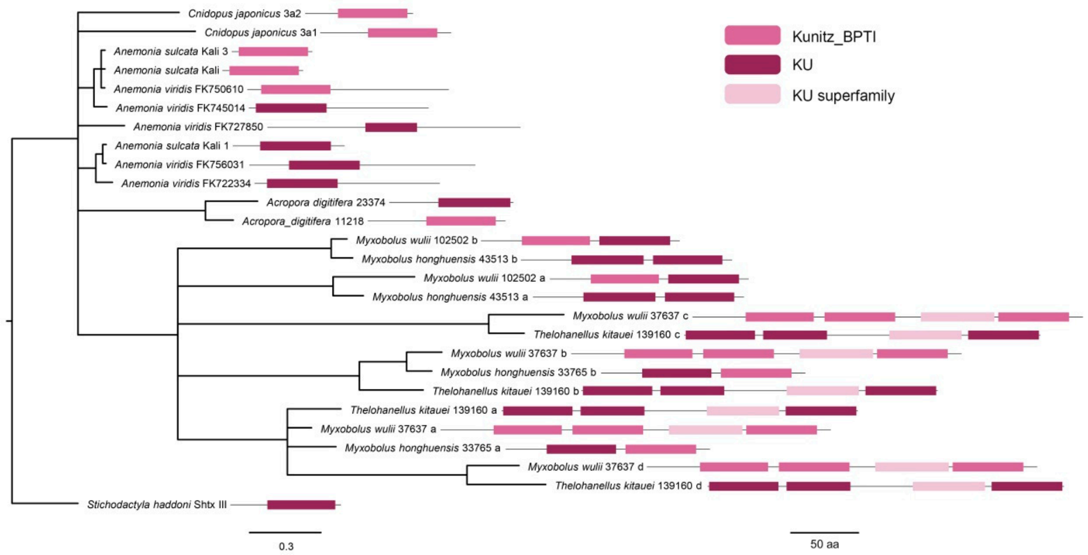


**Figure S15.** Evolutionary relationships of the cnidarian type-2 $K^+$ channel toxins as determined with Bayesian analysis. ShTx-III from *Stichodactyla haddoni* used as an outgroup. Multi-domains in the same proteins were named alphabetically.

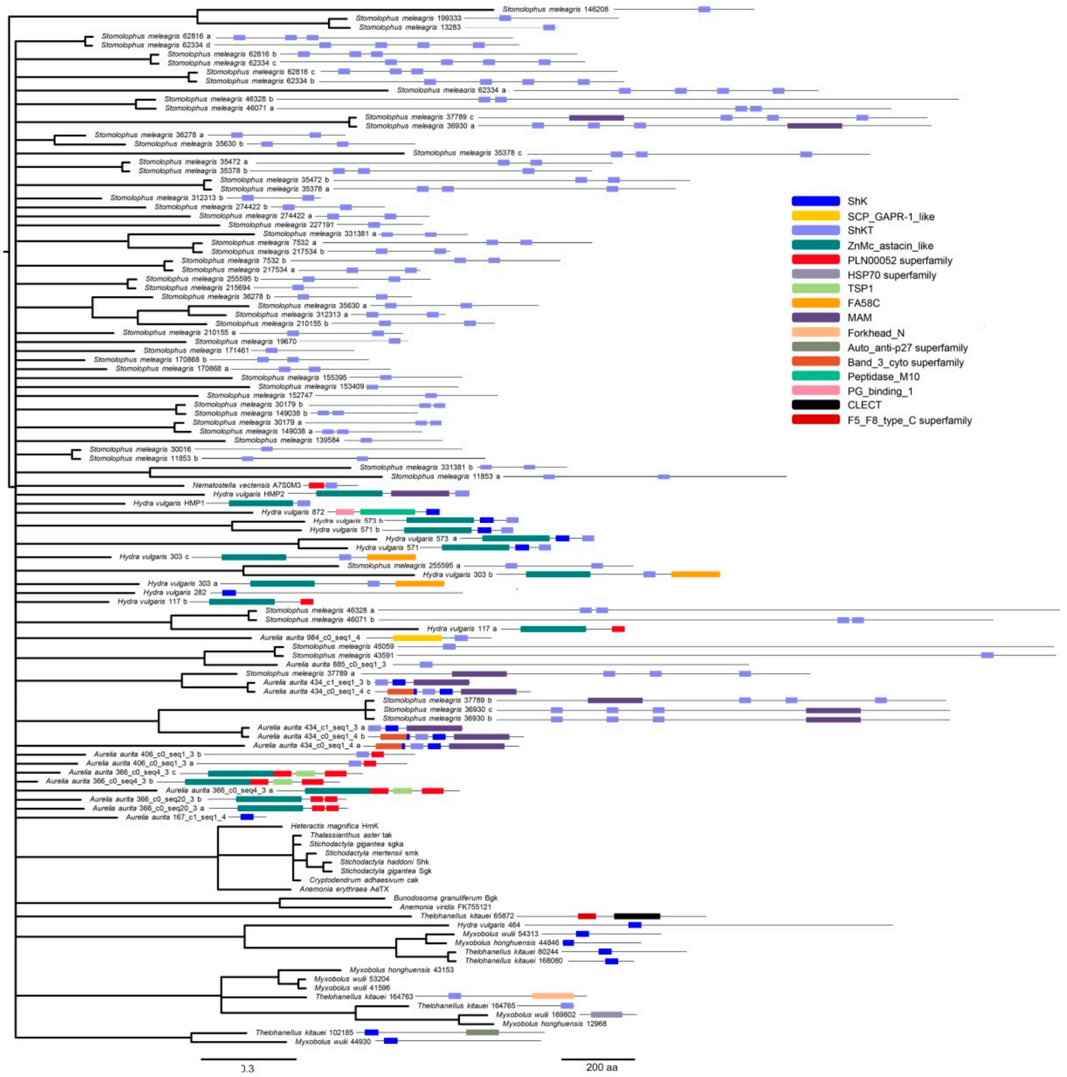


**Figure S16.** A Mid-point-rooted Bayesian tree of the ShK-like toxin domains. Multi-domains in the same proteins were named alphabetically.

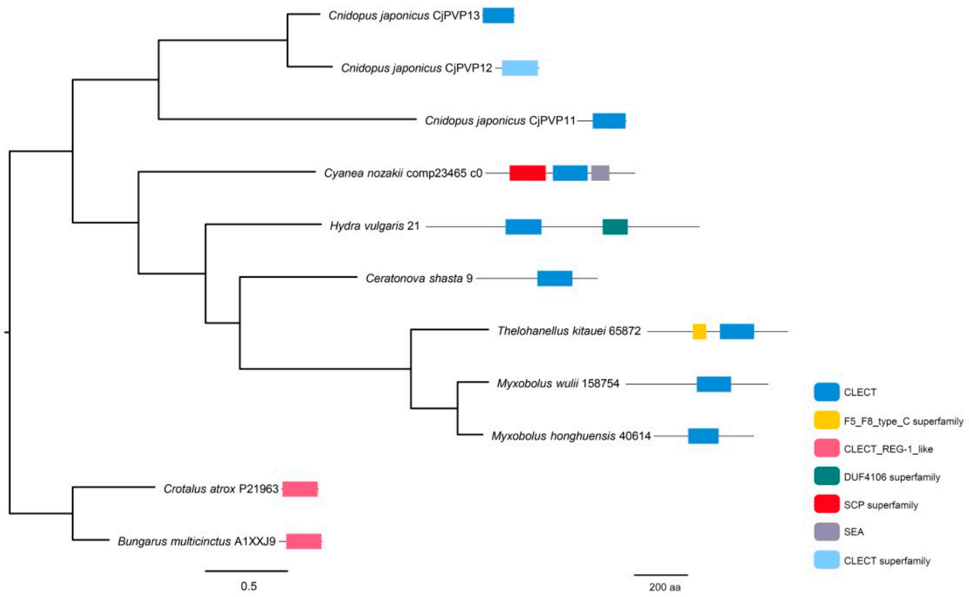


**Figure S17.** Evolutionary relationships of the cnidarian C-type-lectin domain as determined with Bayesian analysis. C-type-lectins from *Crotalus atrox* and *Bunagrus multicinctus* were used as outgroup.

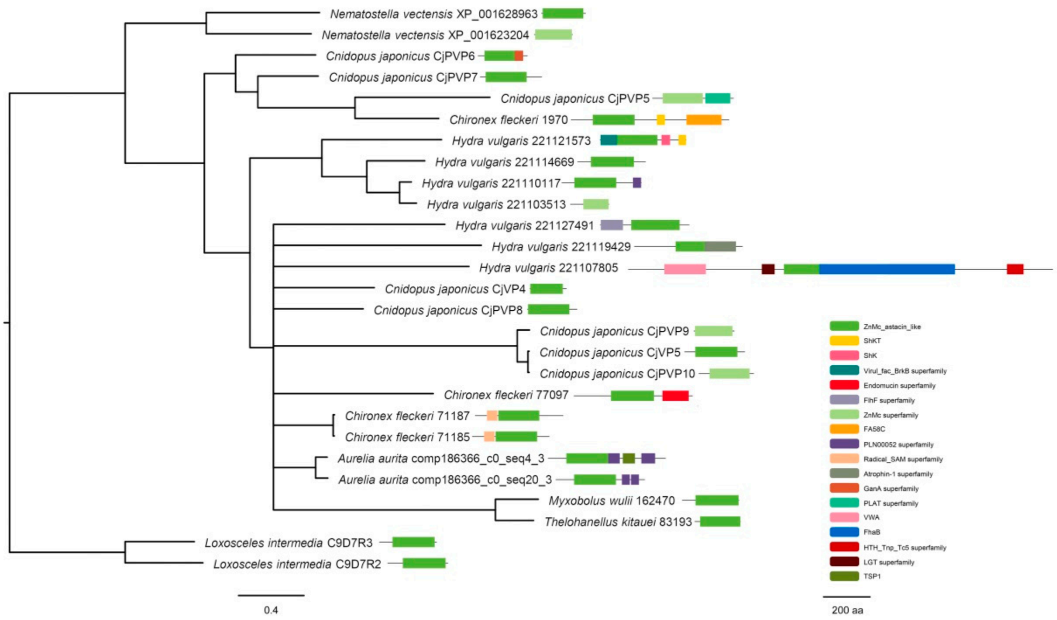


**Figure S18.** Evolutionary relationships of the cnidarian astacin (M12A metalloproteinases) as determined with Bayesian analysis. Astacins from *Loxosceles intermedia* were used as outgroup.

**Table S1.** Sequencing and assembly statistics for genomes and transcriptomes in this study.

| | ***Myxobolus honghuensis*** | | ***Myxobolus wulii*** | | ***Myxobolus ampullicapsulatus*** | ***Myxobolus xiantaoensis*** | ***Myxobolus turpisrotundus*** | ***Thelohanellus kitauei*** | ***Henneguya yanchengensis*** |
|---|---|---|---|---|---|---|---|---|---|
| | **Genome** | **Transcriptome** | **Genome** | **Transcriptome** | **Transcriptome** | **Transcriptome** | **Transcriptome** | **Transcriptome** | **Transcriptome** |
| Raw reads | 160,296,870 | 218,132,320 | 64,872,175 | 100,725,174 | 116,539,988 | 211,600,810 | 151,104,598 | 218,635,192 | 44,201,224 |
| Assembled scaffold /contigs | 384,835 | 27,502 | 629,551 | 42,948 | 276,231 | 98,837 | 171,878 | 90,775 | 378,179 |
| Longest sequence | 130,643 | 12,166 | 26,052 | 12,846 | 17,422 | 11,293 | 18,726 | 13,816 | 13,057 |
| Average length | 791 | 601 | 365 | 1,121 | 390 | 733 | 427 | 580 | 396 |
| N50 | 2,759 | 857 | 806 | 1,445 | 394 | 1,204 | 715 | 1,098 | 399 |
| G-C content | 27.41% | 35.92% | 28.27% | 29.49% | 44.92% | 35.31% | 32.08% | 38.24% | 43% |
| Total assembly size | 205Mb | 16Mb | 260Mb | 47Mb | 108Mb | 71Mb | 75Mb | 53Mb | 150Mb |
| # sequence > 1kb | 66,139 | 4,244 | 49,969 | 18,668 | 12,086 | 22,676 | 16,290 | 14,489 | 15,740 |

0
